# Deciphering the Impact of RAC1‐SPTAN1 in ARPKD Cystogenesis Using Multifaceted Models

**DOI:** 10.1002/advs.202524001

**Published:** 2026-02-26

**Authors:** Shohei Kuraoka, Yuhei Higashi, Suguru Saito, Solmaz Pourgonabadi, Honami Honjoh, Sho Ishigaki, Peter C. Harris, Lisa M. Satlin, Michifumi Yamashita, Ryuji Morizane

**Affiliations:** ^1^ Division of Nephrology Massachusetts General Hospital Harvard Medical School Boston Massachusetts USA; ^2^ Department of Pathology and Laboratory Medicine Cedars‐Sinai Medical Center Los Angeles California USA; ^3^ Division of Nephrology and Hypertension Mayo Clinic Rochester Minnesota USA; ^4^ Department of Pediatrics Icahn School of Medicine at Mount Sinai New York New York USA; ^5^ Harvard Stem Cell Institute Cambridge Massachusetts USA

**Keywords:** kidney, nephron, organoid, organ‐on‐chip, polycystic kidney disease, RAC1, SPTAN1

## Abstract

Autosomal recessive polycystic kidney disease (ARPKD) leads to severe renal cysts and progressive kidney dysfunction, with no approved treatments. The absence of such cystic phenotypes in Pkhd1^−^/^−^ mice underscores the need for novel models that better recapitulate the human disease. We developed kidney organoid‐on‐chip models that mimic patients’ distal‐nephron cysts, identifying RAC1/c‐FOS as potential therapeutic targets. However, critical questions remain regarding RAC1 activation during cyst formation, cyst origins, and underlying molecular mechanisms. Using a multifaceted approach, organoid‐on‐chip models, transgenic mice, and patient kidney samples, we identified reduced levels of SPTAN1, a cytoskeletal spectrin protein, as a key regulator of RAC1 activation and cystic pathology. SPTAN1‐mutant kidney organoids and mice exhibited distal‐nephron cysts, and elevated RAC1/c‐FOS expression, consistent with ARPKD patients. Transcriptomics and live imaging revealed altered calcium signaling and increased intracellular calcium. Single‐cell RNA‐seq identified SLC8A1, a sodium/calcium exchanger, as a marker distinguishing distal/connecting tubules from collecting ducts in human kidneys, predominantly expressed in cystic epithelia in organoids and human ARPKD kidneys. Restoring SPTAN1 in PKHD1^−^/^−^ organoids via CRISPR activation alleviated cystic phenotypes, normalized intracellular calcium, and reduced RAC1/c‐FOS expression. These findings position SPTAN1 as a central player in ARPKD pathogenesis and highlight epigenome editing as a potential therapeutic strategy.

## Graphical abstract

Distal/connecting tubules expressing SLC8A1 have been suggested as a potential origin of ARPKD cysts. SPTAN1 has been identified as a key molecule in ARPKD cyst formation. Restoring SPTAN1 in PKHD1^−^/^−^ organoids reduced cyst formation, normalized calcium levels, and decreased RAC1/c‐FOS expression, highlighting SPTAN1's role in ARPKD and the potential of epigenome editing as a therapeutic approach.

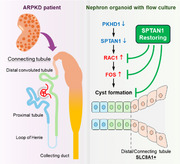

## 1 Introduction

Autosomal recessive polycystic kidney disease (ARPKD) is a severe pediatric ciliopathy characterized by renal cyst formation and progressive kidney dysfunction. Polycystic kidney and hepatic disease 1 (PKHD1) is the causative gene for typical ARPKD, and mutations in both alleles of PKHD1 result in high neonatal mortality and the need for renal replacement therapy in childhood [1]. Despite the identification of PKHD1 as the causative gene, no FDA‐approved drugs exist to slow disease progression. One major challenge in developing treatments is the lack of suitable ARPKD rodent models; Pkhd1^−^/^−^ mouse models often do not develop cysts, and when they do, cyst progression occurs slowly in the proximal tubule segments, highlighting significant species differences [1, 2]. PCK rats, commonly used to model ARPKD, develop cysts in distal nephrons, but the cyst progression is slow and focal, resembling human autosomal dominant PKD (ADPKD) rather than ARPKD [3]. Thus, there is an urgent need to establish an appropriate experimental model that accurately recapitulates ARPKD‐associated renal cysts to enhance our understanding of its pathomechanisms and facilitate therapeutic development.

Recent advances in stem cell research have enabled the generation of kidney organoids from human embryonic stem cells (hESCs) and induced pluripotent stem cells (hiPSCs) [4, 5]. These organoids have been increasingly used to model hereditary renal diseases and are emerging as promising alternatives for diseases that are difficult to reproduce in mice [6, 7]. Although nephron organoids derived from iPSCs established from ARPKD patients have been used to recapitulate cyst pathology, several challenges remain. For instance, cyst formation was dependent on cAMP activators, and the cysts themselves originated primarily from proximal tubules, whereas clinical ARPKD is characterized by cysts predominantly arising from the distal nephron [8]. Notably, cAMP‐activating drugs such as forskolin, which are commonly used to model PKD pathogenesis, have been shown to induce cyst formation in nephron organoids and ex vivo mouse kidneys, even in the absence of genetic mutations [9, 10]. Therefore, the development of a cAMP‐independent PKD disease model is crucial for elucidating the underlying pathomechanism.

Fibrocystin, the protein encoded by PKHD1, is localized in primary cilia and cellular membranes, both of which play crucial roles in mechanosensing. Understanding this aspect is vital to unraveling ARPKD pathology. To explore the mechanosensing pathomechanisms of ARPKD, we developed experimental models specifically designed to examine the effects of mechanical stress on human nephron organoids and cystogenesis [11]. Using fluidic culture, we successfully recapitulated cystic pathology in nephron organoids derived from PKHD1^−^/^−^ ESCs on a millifluidic chip without any cAMP‐activating drugs. Furthermore, our prior study identified elevated RAC1/cFOS signaling in PKHD1^−^/^−^ nephron organoids and demonstrated that targeting this pathway could suppress the cystic phenotype, highlighting potential therapeutic avenues for ARPKD. However, the precise molecular mechanisms by which RAC1 signaling contributes to ARPKD cystogenesis remain unclear. In vivo validation using alternative transgenic models and patient kidney samples is necessary to complement organoid findings. The cellular origin of cysts in ARPKD remains a topic of ongoing debate, particularly in light of results from kidney organoids lacking collecting ducts. Thus, multifaceted strategies and further molecular characterization are essential to understand the pathomechanisms underlying ARPKD cyst formation and to develop targeted therapies.

This study aims to elucidate the mechanisms underlying RAC1 activation in ARPKD cyst formation while recapitulating the disease pathogenesis using a fluidic culture system. Through detailed analyses of the reconstructed cysts, we also investigate their cellular origin and the associated molecular pathways. These findings, including a deeper understanding of the molecular processes involved in ARPKD cystogenesis, are expected to contribute to the development of novel therapeutic strategies for ARPKD.

## 2 Results

### 2.1 Recreating ARPKD Pathophysiology Using a Microfluidic Organoid‐on‐Chip Model With Precision Perfusion Control

**FIGURE 1:**
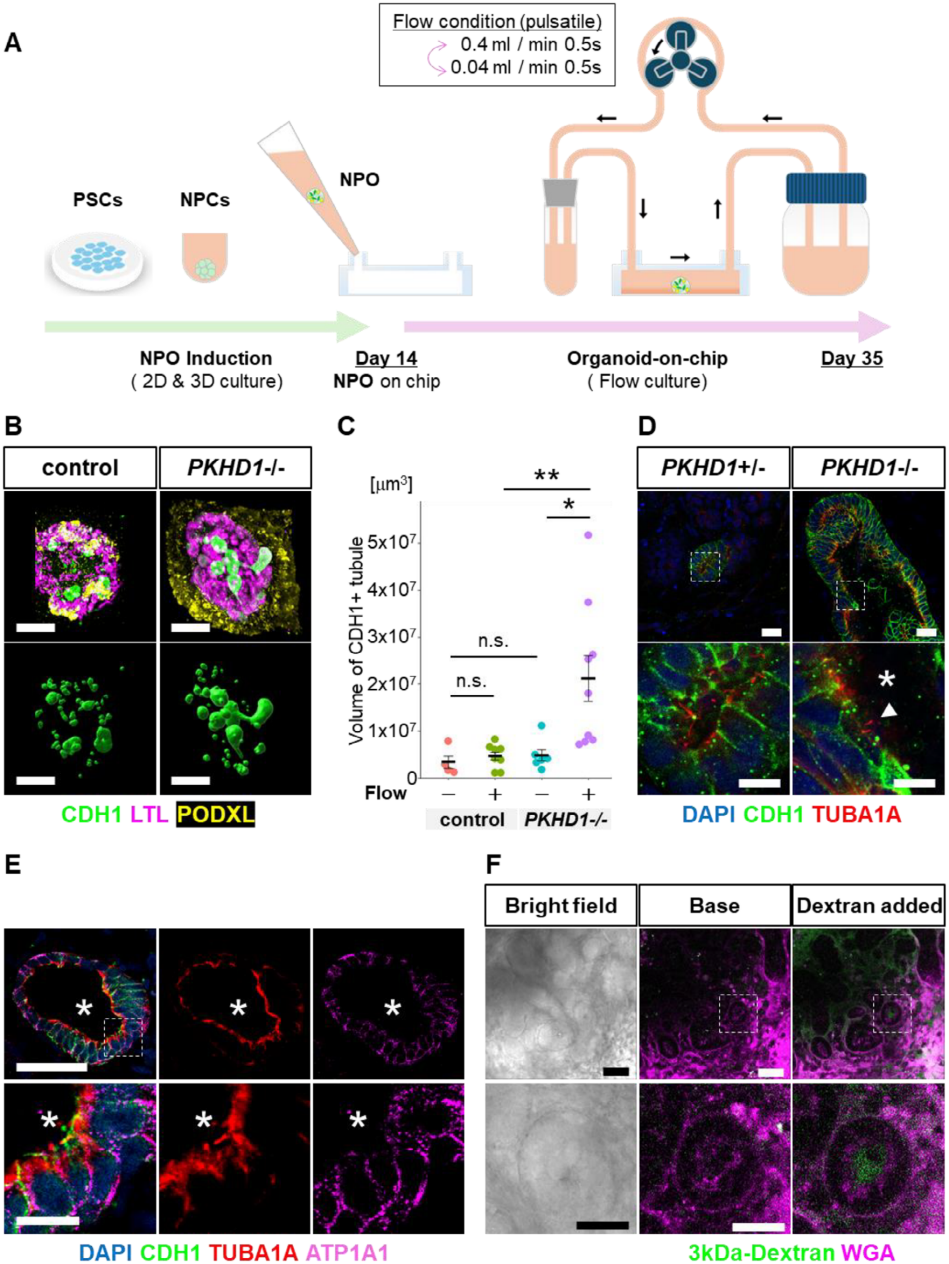
Recreating ARPKD pathophysiology using a microfluidic organoid‐on‐chip model. (A) Schematic representation of a novel organoid‐on‐chip model utilizing a microfluidic chip. PSC, pluripotent stem cell; NPC, nephron progenitor cell; NPO, nephron organoid. (B) Upper panels: Whole‐organoid 3D confocal imaging stacks of fluidic‐cultured PKHD1^+^/^−^ and PKHD1^−^/^−^ organoids on day 35. Lower panels: Imaris surface for CDH1. The yellow signal observed outside corresponds to non‐specific background from Geltrex. Scale bars: 200 µm. (C) Quantification of the total tubule volume in nephron organoids on day 35. The left panel shows the results for CDH1+ tubules. Each dot represents the value of a single organoid. Each condition contains 5–9 organoids. **p < 0.01, *p < 0.05, n.s.: not significant. (D) Immunostaining for the ciliary marker TUBA1A in fluidic‐cultured PKHD1^+^/^−^ and PKHD1^−^/^−^ organoids on day 35. Asterisks indicate dilated lumens, while arrowheads mark primary cilia. Scale bars: upper panels, 50 µm; lower panels, 10 µm. (E) Immunostaining for the ciliary marker TUBA1A and the basolateral marker ATP1A1 in fluidic‐cultured kidney organoids on day 35. Asterisks indicate apical side. Scale bars: upper panels, 50 µm; lower panels, 10 µm. (F) Live perfusion imaging of 3kDa‐Dextran under pulsatile flow conditions (0.4 and 0.04 mL min^−1^ every 0.5 s). The lumens of WGA+ tubules are filled with dextran, consistent with tubular luminal flow. Scale bar: 100 µm.

Prior to investigating the role of RAC1 in ARPKD, we optimized our previously reported flow culture system [11, 12] (Figure 1A). The new organoid‐on‐chip model utilizes commercially available microfluidic chips and incorporates a programmable perfusion pump, replacing 3D‐printed millifluidic chips and the peristaltic pump used in the prior system. Kidney organoids derived from PSCs were transferred into microfluidic chips on day 14, at which point they no longer required any growth factors for further differentiation. After overnight incubation at 37°C, the organoids were exposed to medium flow from day 15 to day 35. The use of commercially available microfluidic chips enabled precise control of shear stress by adjusting flow speed. These modifications allowed for the application of a defined shear stress of 0.1 dyn/cm^2^ to nephron organoids at a medium flow rate of 0.4 mL/min, compared to the previous system's 0.01 dyn/cm^2^ at 4.27 mL/min. The applied shear stress of 0.1 dyn/cm^2^ closely approximates physiological levels observed in the kidney [13, 14, 15], although these forces correspond to luminal flow within nephrons, whereas our setup applies shear stress to the peritubular (basolateral) surface of the organoid.

In vivo, glomeruli filter blood from afferent arterioles into the proximal tubules, where tubular luminal flow is also considered pulsatile. To replicate this physiological condition, our organoid‐on‐chip model applies a pulsatile medium flow, alternating between 0.4 and 0.04 mL/min every 0.5 s. The peristaltic pump used in our previous study had the limitation of generating pulsatile flow only at high flow rates while producing continuous flow at low flow rates. In contrast, the current pump system allows precise control, enabling the selection of either pulsatile or continuous flow at any desired flow rate. By culturing PKHD1^−^/^−^ nephron organoids under flow conditions from day 15 to day 35, we successfully reproduced distal nephron cysts without forskolin treatment (Figure 1B). Cyst‐like tubule dilation was flow‐dependent and specific to CDH1‐positive distal nephrons, consistent with ARPKD patients (Figure 1C; Figure S1). Additionally, primary cilia localized to the apical side of dilated distal nephrons in PKHD1‐/‐ organoids (Figure 1D). Furthermore, ATP1A1, a marker of distal tubules, was expressed on the basolateral side of these structures, indicating that apical–basal polarity is preserved in the organoids (Figure 1E). To assess flow within organoids, we introduced 3 kDa dextran into the medium reservoir and performed live imaging under flow conditions in organoids labeled with wheat germ agglutinin (WGA), a lectin that binds to nephron epithelia. Dextran was simultaneously detected in both the tubular lumen and interstitial space during live imaging, consistent with the presence of both luminal and interstitial flow within the organoids (Figure 1F; Figure S2A,B). While, in static conditions, dextran was detected only in the interstitial space, not in the tubular lumen (Figure S2C). Furthermore, a linear mixed‐effects model revealed that the rate of increase in intensity was significantly higher in the flow condition than in the static condition (estimate = 0.44, p = 2.0 × 10^−^^8^) (Figure S2D). These findings suggest that the observed movement is not due to passive diffusion or apical uptake. These improvements enhanced the model's applicability, regulation, and reproducibility. This modified organoid‐on‐chip model was used in subsequent experiments.

### 2.2 Decline of the RAC1 Partner, SPTAN1, in ARPKD Organoids and Patient Cysts

**FIGURE 2:**
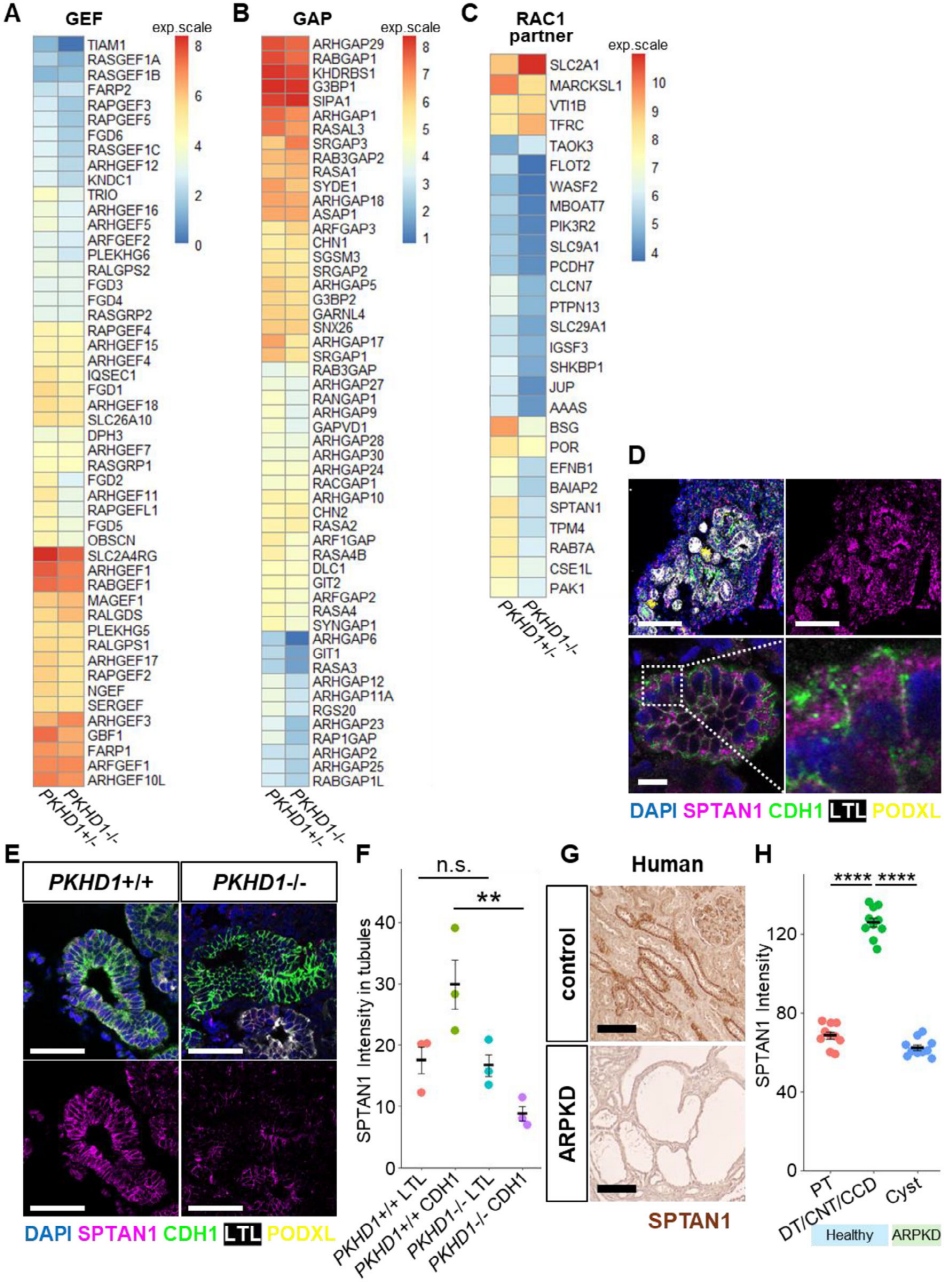
Decline of the RAC1 partner, SPTAN1, in ARPKD. (A–C) Heat maps of GEF (A), GAP (B), and the potential RAC1 partner genes (C), generated from microarray data comparing fluidic‐cultured PKHD1^+^/^−^ and PKHD1^−^/^−^ organoids. exp.scale: expression scale. (D) Immunostaining for SPTAN1, CDH1, LTL, and PODXL in fluidic‐cultured wild‐type nephron organoids on day 35. Scale bars: upper panels, 100 µm; lower panels, 10 µm. (E) Immunostaining for SPTAN1, CDH1, LTL, and PODXL in fluidic‐cultured PKHD1^+^/^−^ and PKHD1‐/‐ organoids at day 35. Scale bars: 50 µm. (F) Quantification of the SPTAN1 intensity in LTL+ and CDH1+ tubules. Each dot represents the mean value of a single organoid. Each condition contains 3 organoids on day 35. **p < 0.01. (G) Immunohistochemistry for SPTAN1 in human kidneys of healthy controls and ARPKD patients. Scale bars: 100 µm. (H) Quantification of the SPTAN1 intensity in human samples. Each dot represents the value of a single tubule. Each segment contains 10 tubules or cysts from one ARPKD patient. ****p < 0.0001. PT, proximal tubule; DT, distal tubule; CNT, connecting tubule; CCD, cortical collecting duct.

Our previous study demonstrated increased active RAC1 expression both in ARPKD organoids cultured under medium flow and in kidney tissues from ARPKD patients, and we reported an improvement in cystic manifestations with three different drugs that inhibit RAC1 [11]. This suggests that elevated RAC1 levels are causative for ARPKD; however, it remains unclear how RAC1 activation is enhanced in ARPKD. From our microarray analysis comparing PKHD1^+^/^−^ and PKHD1^−^/^−^ nephron organoids cultured under flow conditions, we found that while guanine nucleotide exchange factors (GEFs; RAC1 activators) such as TIAM1 and TRIO were downregulated, no significant differences were observed in GTPase‐activating proteins (GAPs; RAC1 deactivators) (Figure 2A,B). This was thought to represent a negative feedback response to RAC1 hyperactivation. The Rho family of GTPases, including RAC1, is known to interact with effector proteins [16], and RAC1 downstream processes can be influenced by these effectors and their expression levels. Thus, we hypothesized that the elevated active RAC1 levels in ARPKD might result from an alteration in partner molecules. To assess RAC1 effector expression, we analyzed gene lists of potential partners of major Rho GTPases, identified based on protein proximity interactions in a human fetal kidney cell line [17]. By comparing the expression of these partners with differentially expressed genes (DEGs) between fluidic‐cultured PKHD1^−^/^−^ and PKHD1^+^/^−^ organoids on day 35, we identified 27 altered RAC1 partners, most of which were downregulated in PKHD1^−^/^−^ organoids (Figure 2C). These findings suggest that the elevation of active RAC1 in cystic epithelia is likely due to the downregulation of RAC1 partner proteins. Notably, these 27 partners included molecules involved in transporters and cell adhesion. Considering previous studies and findings in human kidneys, we identified SPTAN1, coding α‐II‐spectrin, as a candidate gene potentially involved in the cystic biological process. Spectrin is a core cortical scaffold that forms a membrane‐proximal lattice regulating the access of cytosolic proteins to submembrane regions, including small GTPase regulators such as GEFs and GAPs. Consistent with this, spectrin depletion has been reported to activate RAC1 [18]; therefore, we hypothesized that SPTAN1 reduction increases RAC1 activity and thereby accelerates cyst formation in ARPKD.

SPTAN1 is a causative gene for the infantile‐onset epileptic encephalopathy type 5, also referred to as SPTAN1 encephalopathy [19, 20]. While most in‐frame and missense mutations in SPTAN1 are associated with severe epilepsy, some mutations are not, and the clinical manifestations of SPTAN1 mutations vary depending on the mutation type [19]. Notably, a case report described a patient with a heterozygous SPTAN1 nonsense mutation who presented with renal cysts at the fetal stage, along with microcephaly, global developmental delay, and multiple congenital anomalies, but without severe epilepsy [21]. SPTAN1 is known to play a role in actin‐cytoskeleton regulation and potentially in the cell cycle [22, 23]. Our immunostaining for SPTAN1 revealed particularly strong expression in distal nephrons, with lower ubiquitous expression in kidney organoids (Figure 2D). SPTAN1 was localized just beneath the plasma membrane in tubules, consistent with its role as a scaffold protein. In PKHD1^−^/^−^ nephron organoids cultured under flow conditions on day 35, SPTAN1 expression was significantly downregulated in CDH1‐positive distal nephrons, not in LTL‐positive proximal tubules (Figure 2E,F). Immunostaining of human kidney tissues showed that SPTAN1 was strongly expressed in non‐proximal tubules, morphologically corresponding to distal tubules, connecting tubules, and cortical collecting ducts in healthy controls. In contrast, SPTAN1 expression was reduced in the renal cystic epithelium of ARPKD patients, consistent with findings in PKHD1^−^/^−^ nephron organoids (Figure 2G,H). In these assessments, microarray analysis and immunostaining were performed on different batches of nephron organoids, and the consistency of these results with patient samples supports the reproducibility of our findings. Collectively, these results suggest that reduced SPTAN1 expression may be involved in cystic pathological processes in ARPKD.

### 2.3 Genetic Confirmation of Cysts Caused by SPTAN1 Reduction in Transgenic Human Organoids and Mice

To investigate the role of SPTAN1 in cystogenesis, first, we established heterozygous and homozygous mutant hPSC lines for the SPTAN1 gene using CRISPR/Cas9 gene editing as we described previously [24]. Transduction of the CRISPR vector by Lipofection resulted in a single nucleotide insertion in exon 1, and this mutation causes a frameshift and the absence of the full‐length SPTAN1 protein; immature stop codon (p.Phe29fs*8, Figure S3A). The SPTAN1^+^/^−^ hESC lines were capable of differentiating into nephron organoids under the same conditions as the parental ESC line, whereas the SPTAN1^−^/^−^ ESC lines failed to differentiate. SPTAN1^+^/^−^ nephron organoids were further cultured under flow conditions from day 15 to day 35, using the organoid‐on‐chip system. On day 35, we performed whole‐mount staining for SPTAN1^+^/^+^ and SPTAN1^+^/^−^ nephron organoids and quantified the following: total glomerular volume (PODXL‐positive), total proximal tubular volume (LTL‐positive), and total distal nephron volume (CDH1‐positive) using 3D reconstruction.

**FIGURE 3:**
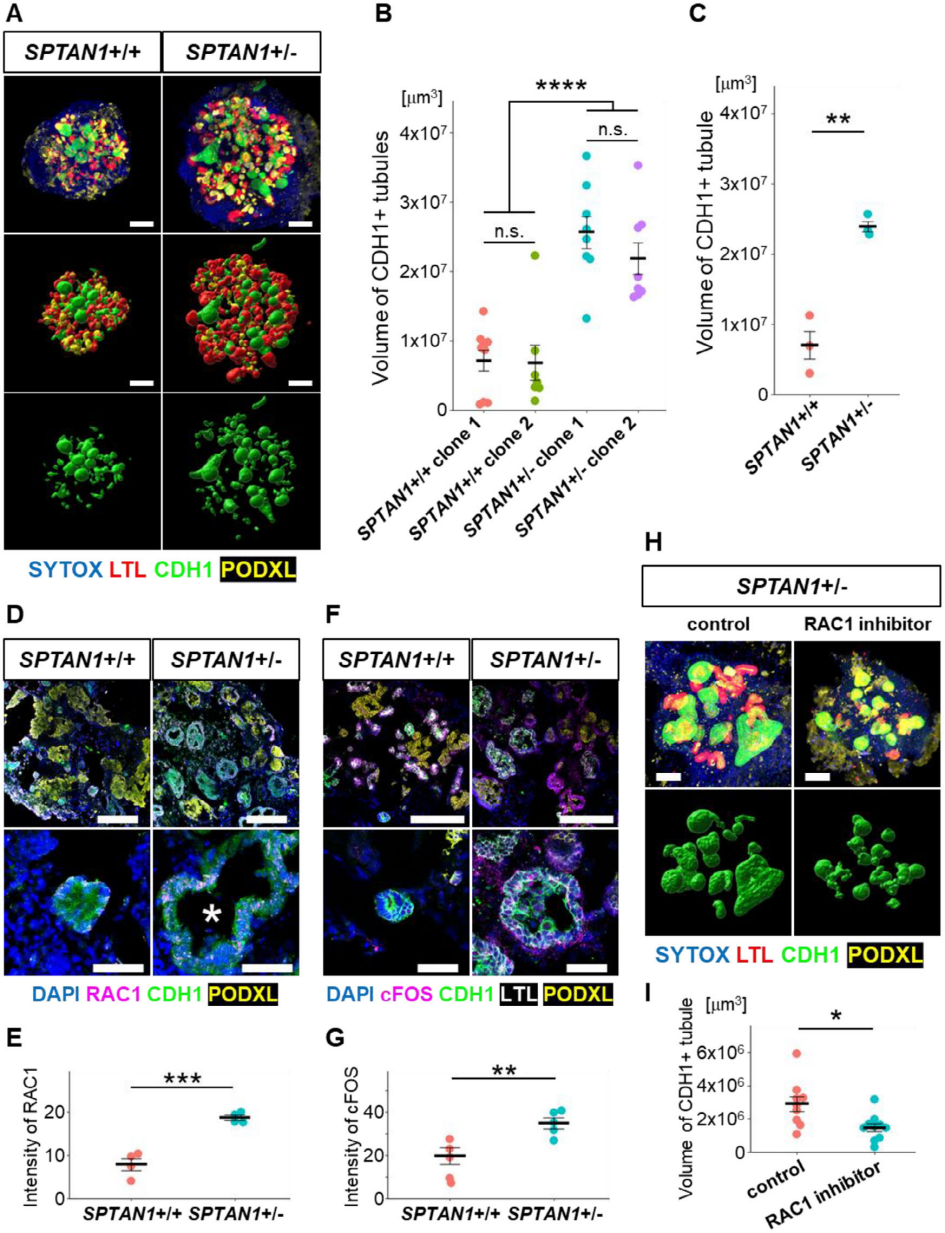
Genetic confirmation of cysts caused by SPTAN1 reduction. (A) 3D imaging of fluidic cultured SPTAN1^+^/^+^ and SPTAN1^+^/^−^ organoids on day 35. Scale bars: 200 µm. (B) Quantification of the total CDH1+ tubule volume on day 35 in each clone cultured under flow conditions. Each dot represents the value of a single organoid. Each condition contains 7–9 organoids respectively. ****p < 0.0001, n.s.: not significant. (C) Quantification of the total CDH1+ tubule volume on day 35 in nephron organoids cultured under flow conditions. Each dot represents the average result of a single independent experiment, which includes 5–6 organoids. Each condition contains 16 organoids. **p < 0.01. (D) Immunostaining for RAC1 in fluidic‐cultured SPTAN1^+^/^+^ and SPTAN1^+^/^−^ organoids on day 35. Asterisks indicate dilated lumens. Scale bars: upper panels, 200 µm; lower panels, 50 µm. (E) Quantification of the RAC1 intensity in CDH1+ tubules. Each dot represents the mean value of a single organoid. Each condition contains 4 organoids. **p < 0.01. (F) Immunostaining for cFOS in fluidic‐cultured SPTAN1^+^/^+^ and SPTAN1^+^/^−^ organoids on day 35. Scale bars: upper panels, 200 µm; lower panels, 50 µm. (G) Quantification of the cFOS intensity in CDH1+ tubules. Each dot represents the mean value of a single organoid. Each condition contains 5 organoids. **p < 0.01. (H) 3D imaging of fluidic cultured SPTAN1^+^/^−^ organoids with the RAC1 inhibitor on day 35. Scale bars: 100 µm. (I) Quantification of the total CDH1+ tubule volume on day 35 in nephron organoids treated with the RAC1 inhibitor (NSC23766) under flow conditions. Each dot represents the value of a single organoid. Each condition contains 9–10 organoids. *p < 0.05.

Importantly, SPTAN1^+^/^−^ nephron organoids exhibited a significant increase in CDH1+ distal nephron volume (Figure 3A–C). To assess the reproducibility of this key finding, we tested two clones in each condition: two heterozygous mutant clones with the same mutation and two control clones obtained during the establishment of the SPTAN1 mutant ES cell line. No significant variation was observed between clones (Figure 3B). On the other hand, no significant differences were observed in the volumes of both proximal tubules and glomeruli, suggesting that the effect of the SPTAN1 mutation is specific to the distal nephron (Figure S3B,C). We also evaluated immunostaining of RAC1 and found a significant upregulation of RAC1 in the CDH1‐positive distal nephron of fluidic‐cultured SPTAN1^+^/^−^ nephron organoids (Figure 3D,E; Figure S3D). Additionally, cFOS expression was also upregulated in the CDH1‐positive distal nephron of SPTAN1^+^/^−^ nephron organoids (Figure 3F,G; Figure S3D). Furthermore, consistent with our prior study showing that the RAC1 inhibitor NSC‐23766 suppressed cyst formation in PKHD1−/− organoids [11], treatment of SPTAN1^+^/^−^ organoids with NSC‐23766 similarly reduced distal nephron dilation (Figure 3H,I). These findings indicate that fluidic‐cultured SPTAN1^+^/^−^ nephron organoids can recapitulate the cystic pathology seen with PKHD1^−^/^−^ nephron organoids cultured under medium flow, suggesting that SPTAN1 plays a crucial role in the cystic biological process through RAC1 activation in ARPKD.

**FIGURE 4:**
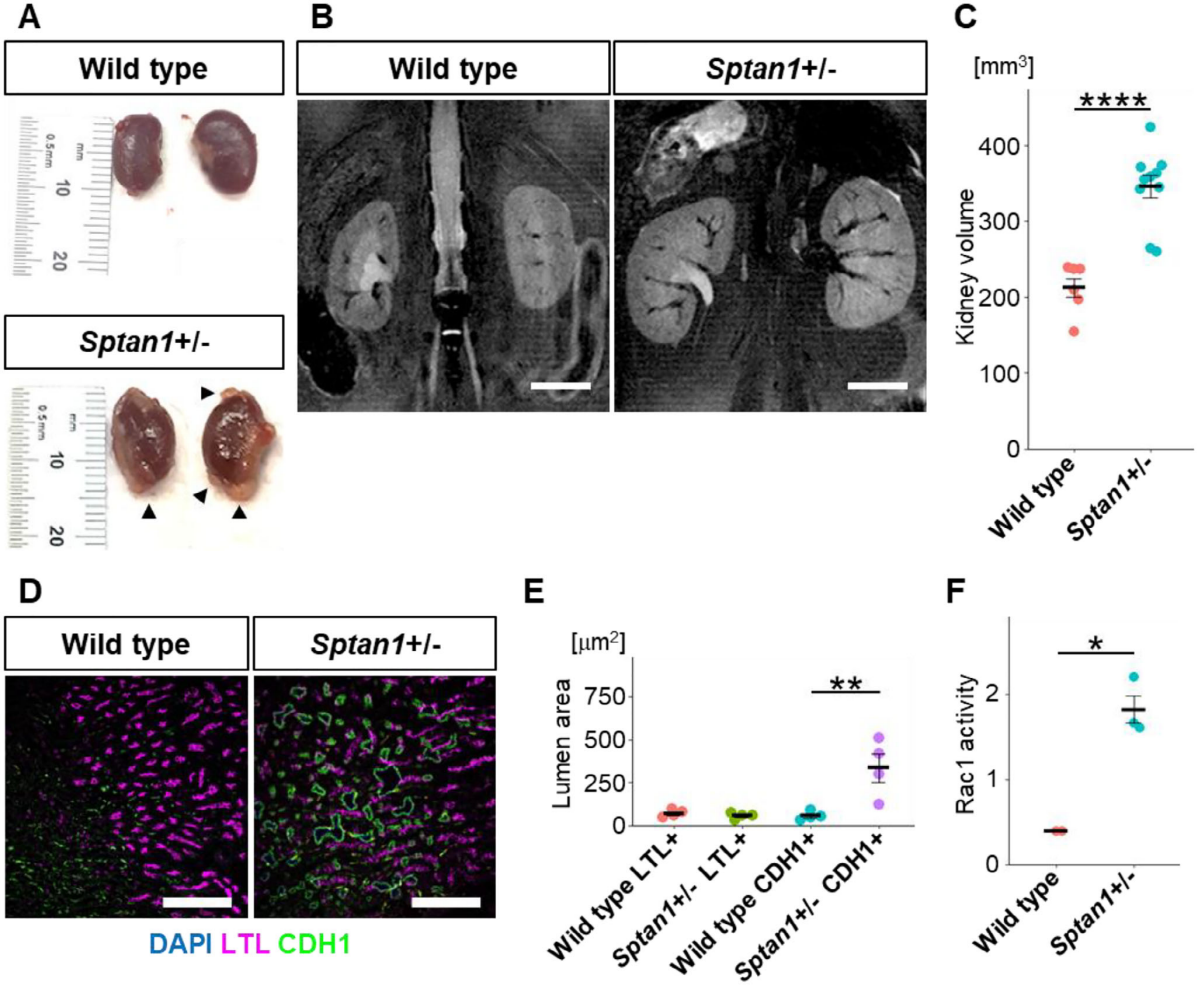
Cystic phenotype of Sptan1 transgenic mice. (A) Appearance pictures of Sptan1^+^/^−^ mouse kidneys at 13 months of age. Arrowheads mark macroscopic cysts. (B) MRI T2 images of mouse kidneys at 1 year of age. Scale bars: 5 mm. (C) Quantification of the volume of kidneys. Each dot represents the value of a single kidney. ****p < 0.0001. (D) Immunohistochemistry for CDH1, LTL, and DAPI in mouse kidney tissues at 16 weeks of age. Scale bars: 200 µm. (E) Quantification of the luminal area in tubules. Each dot represents the mean value of a single kidney. Each condition contains 32, 31, 27 and 36 tubules respectively from 4 kidneys. **p < 0.01. F) Quantification of the Rac1 activity by G‐LISA in kidney tissues. Each dot represents the corrected value of a single kidney. *p < 0.05.

For in vivo validation, we explored Sptan1 mutant mouse models. The International Mouse Phenotyping Consortium (IMPC) demonstrated that Sptan1 heterozygous mutant mice, in which exon 9 and its flanking splicing regions were constitutively deleted using CRISPR/Cas9, exhibited renal enlargement in early adulthood along with multiple ocular morphological abnormalities. At 1 year of age, occasional macroscopic cysts were visible on the kidney surface of Sptan1^+^/^−^ mice but not wild‐type controls (Figure 4A). To confirm and quantify this phenotype, kidney volumes were compared by MRI, which enabled accurate volumetry; Sptan1^+^/^−^ kidneys were significantly enlarged (Figure 4B,C; Figure S4A). To investigate further, we investigated the immunostaining of kidney tissue sections provided by IMPC. The Sptan1^+^/^−^ mice exhibited sporadic microscopic dilations in the CDH1+ distal nephron (Figure 4D,E; Figure S4B). Furthermore, we found that Rac1 activity was increased in the kidney tissue of Sptan1 mutant mice (Figure 4F). These findings support our hypothesis that SPTAN1 contributes to the pathogenesis of ARPKD, including the hyperactivation of RAC1, and suggest that Sptan1 mutant mice may serve as a potential model for studying ARPKD.

### 2.4 SLC8A1‐Positive Connecting Tubules Are the Potential Origin of ARPKD Cysts

**FIGURE 5:**
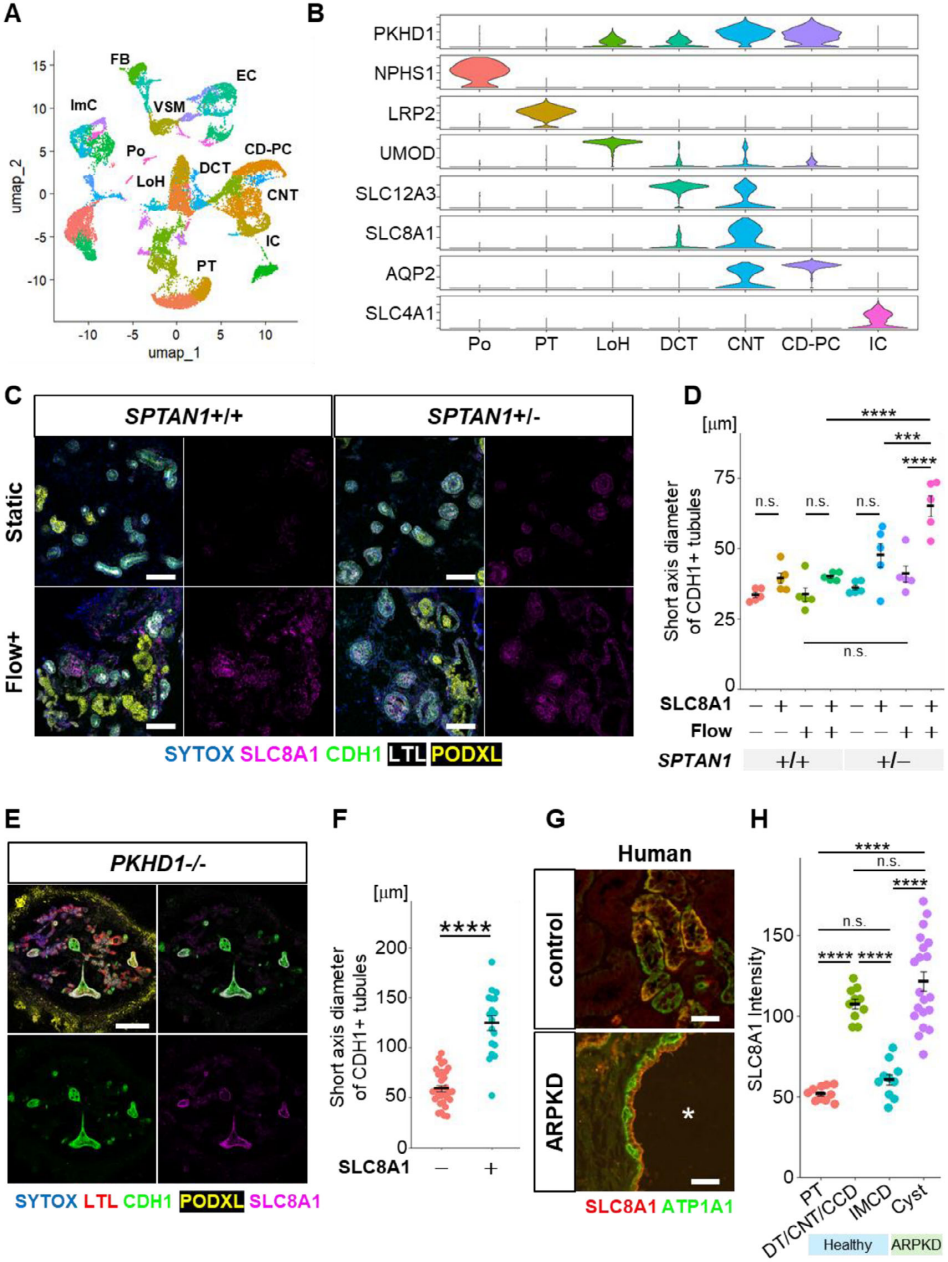
SLC8A1‐positive connecting tubules are the potential origin of ARPKD cysts. (A) UMAP generated from scRNA‐seq of 24 healthy human kidneys. Po, podocytes; PT, proximal tubules; LoH, loop of Henle; DCT, distal convoluted tubules; CNT, connecting tubules; CD‐PC, collecting duct principal cells; IC, intercalated cells; FB, fibroblast cells; EC, endothelial cells; VSM, vascular smooth muscle cells; ImC, immune cells. (B) PKHD1 and signature gene expression of each nephron segment. (C) Immunostaining for SLC8A1 in SPTAN1^+^/^+^ and SPTAN1^+^/^−^ organoids. Upper panels, static condition; lower panels, flow condition. Scale bars: 100 µm. (D) Quantification of the short‐axis diameters of CDH1+ tubules. Each dot represents the mean value of each organoid. Each condition contains 5 organoids. *p < 0.05, n.s.: not significant. (E) Immunostaining for SLC8A1 in fluidic‐cultured PKHD1‐/‐ organoids on day 35. Scale bar: 500 µm. (F) Quantification of the short‐axis diameters of CDH1+ tubules in PKHD1^−^/^−^ organoids. Each dot represents the measurement of an individual tubule. Each condition contains 30 and 18 tubules respectively from 2 organoids. ****p < 0.0001. (G) Immunostaining for SLC8A1 and ATP1A1 in human kidneys of healthy controls and ARPKD patients. Asterisks indicate dilated lumens. Scale bars: 100 µm. (H) Quantification of the SLC8A1 intensity in human samples. Each dot represents the value of a single tubule. Each segment contains 10 tubules or 20 cysts. ****p < 0.0001. PT, proximal tubule; DT, distal tubule; CNT, connecting tubule; CCD, cortical collecting duct; IMCD, inner medullary collecting duct.

To assess the faithfulness of SPTAN1‐mutant models for ARPKD studies, we revisited the origin of cysts in ARPKD. Previous studies have investigated the origin of ARPKD cysts using lectin staining of patient kidneys, revealing PNA positivity but not LTL positivity in cysts [25]. PNA primarily targets distal tubules and a small fraction of cells in collecting ducts, suggesting that ARPKD cysts may arise from both distal tubules and collecting ducts [26, 27]. However, the precise origin of these cysts remains unclear due to limited characterization. Based on observations of cyst development in CDH1+ tubules in nephron organoids, where collecting ducts are absent, we hypothesized that ARPKD cysts primarily originate from distal/connecting tubules. To address this hypothesis, we first analyzed single‐cell RNA sequencing (scRNA‐seq) from 24 percutaneous renal biopsy samples obtained from healthy humans, provided by the Kidney Precision Medicine Project (KPMP) [28]. By integrating data from these large datasets, we classified nephron segments into the following categories based on marker genes identified by the gene list reported previously (Figure S5) [29]: Podocyte, Proximal tubules, Loop of Henle, Distal convoluted tubule (DCT), Connecting tubule (CNT), Collecting duct‐Principal cell (CD‐PC) and Intercalated cells (Figure 5A).

The scRNA‐seq analysis revealed that PKHD1 is strongly expressed in CD‐PC, with equivalent expression detected in CNT (Figure 5B). CNT expresses SLC12A3, a DCT marker, and AQP2, which is characteristic of CD‐PC, exhibiting features of both distal tubules and the collecting duct. However, our scRNA‐seq data showed that SLC8A1 is predominantly expressed in CNT, with lower expression in DCT, and absent in CD‐PC, consistent with previous findings in rats [30]. Immunostaining further confirmed that our induced nephron organoids expressed SLC8A1 in some of the CDH1‐positive distal nephrons, indicating the presence of tubules corresponding to DCT/CNT (Figure 5C). Additionally, wild‐type nephron organoids exhibited a higher percentage of SLC8A1‐positive distal nephrons when cultured under flow conditions using the organoid‐on‐chip system, compared to conventional static culture (Figure S6A). Then, we measured the short‐axis diameters of SLC8A1‐positive and SLC8A1‐negative tubules/cysts in CDH1+ distal nephrons (Figure 5D). In wild‐type organoids, no significant difference in short‐axis diameter was observed between SLC8A1‐positive and SLC8A1‐negative tubules, and this pattern persisted under flow culture. However, in SPTAN1^+^/^−^ nephron organoids, the short‐axis diameter of SLC8A1‐positive tubules was significantly enlarged, while the diameter of SLC8A1‐negative distal nephrons remained similar to controls. Similarly, PKHD1^−^/^−^ nephron organoids also exhibited enlargement of CDH1+/SLC8A1+ distal nephrons, mirroring the results seen in SPTAN1^+^/^−^ nephron organoids (Figure 5E,F). Furthermore, in healthy human kidneys, SLC8A1 was colocalized with ATP1A1‐positive distal nephron segments, including the distal tubules (DT), connecting tubules (CNT), and cortical collecting ducts (CCD), but not with LTL+ proximal tubules (Figure 5G; Figure S6B). The inner medullary collecting ducts (IMCD) exhibited expression levels comparable to those of the proximal tubules (Figure 5H; Figure S6C). Importantly, kidneys from ARPKD patients showed that the majority of cysts were positive for SLC8A1, with intensity levels similar to those detected in the cortical distal nephron (Figure 5G,H). These findings suggest that ARPKD cysts primarily originate from SLC8A1‐positive distal nephron segments, mainly consist of DT and CNT.

Moreover, we performed immunostaining of kidney sections from Sptan1 mutant mice using SLC8A1. In both wild‐type and Sptan1^+^/^−^ kidneys, SLC8A1 expression was predominantly observed in distal tubules located at the corticomedullary junction. In Sptan1^+^/^−^ kidneys, dilation of distal tubule lumens at the corticomedullary junction was readily apparent even at low magnification, and these dilated tubules were clearly SLC8A1‐positive (Figure S7A). This dilation was also evident by quantitative analysis of luminal area. In contrast, distal tubules located in the cortex that were negative for SLC8A1 did not show an obvious difference between wild‐type and SPTAN1+/− kidneys (Figure S7B). These results indicate that Sptan1^+^/^−^ mice exhibit a similar phenotype in SLC8A1‐positive distal nephron segments.

### 2.5 SPTAN1 Mutation Alters Calcium Signaling in Fluidic Cultured Organoids

**FIGURE 6:**
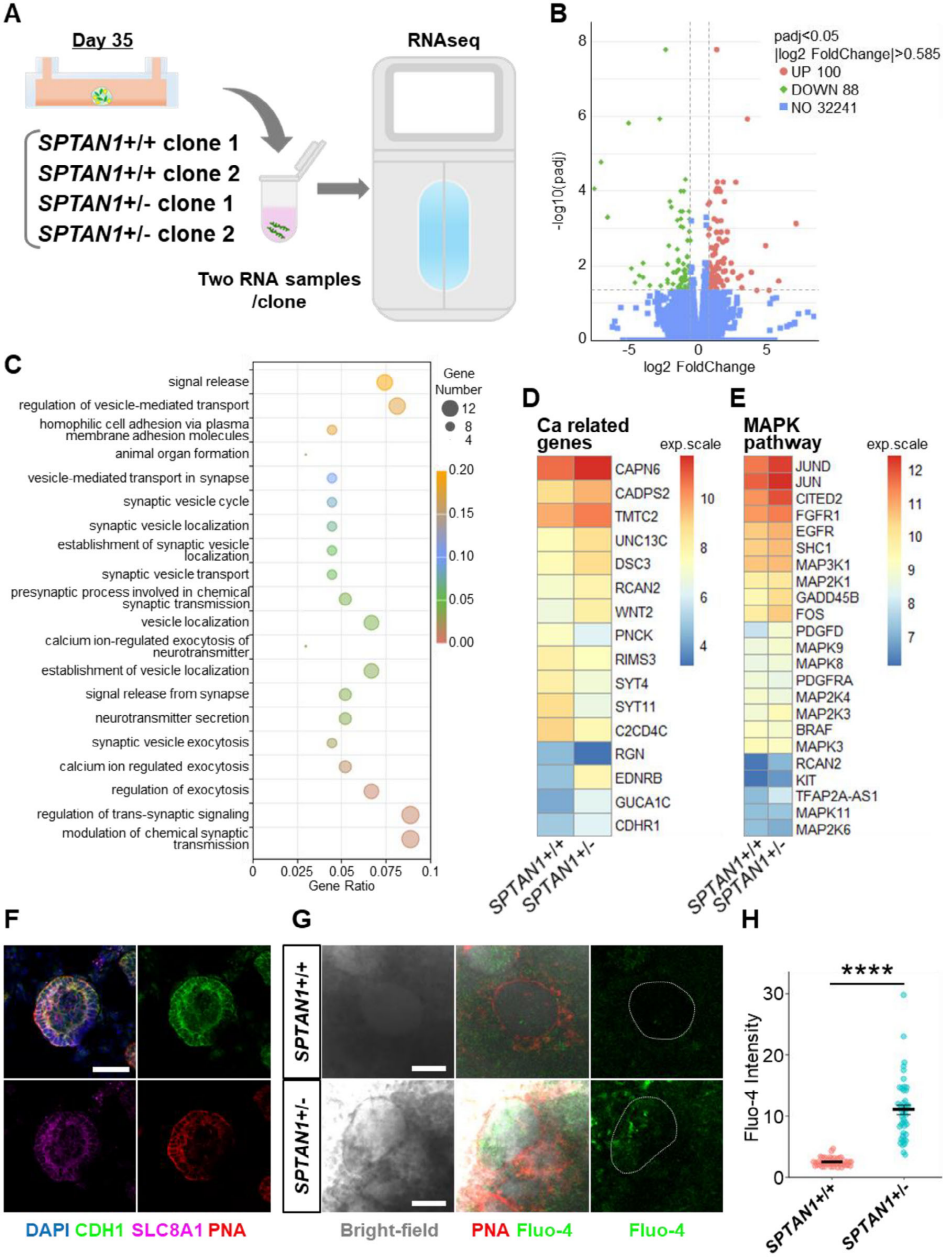
Altered calcium signaling by SPTAN1 mutation. (A) Schematic representation of RNA‐seq analysis utilizing day 35 nephron organoids in a microfluidic chip. RNA samples were extracted from two SPTAN1^+^/^+^ clones and two SPTAN1^+^/^−^ clones. (B) Volcano plot of gene expression in SPTAN1^+^/^+^ and SPTAN1^+^/^−^ organoids. (C) Biological processes in GO analysis generated from the DEG list between SPTAN1+/+ and SPTAN1^+^/^−^ organoids. (D) Heat map of calcium‐related genes generated from the DEG list comparing fluidic‐cultured SPTAN1^+^/^+^ and SPTAN1^+^/^−^ organoids. (E) Heat map of MAPK pathway‐related genes comparing fluidic‐cultured SPTAN1^+^/^+^ and SPTAN1^+^/^−^ organoids. (F) Immunostaining for SLC8A1 and PNA in SLC8A1^+^/^+^ organoids. Scale bar: 50 µm. (G) Ca imaging by Fluo‐4 in fluidic‐cultured SPTAN1+/+ and SPTAN1^+^/^−^ organoids on day 35. Scale bars: 50 µm. (H) Quantification of the Fluo‐4 intensity in PNA+ tubules. Each dot represents the mean value of a single epithelial cell. Each condition contains 45 epithelial cells from 3 organoids. ****p < 0.0001.

To elucidate the cystic biological processes in SPTAN1 mutants, we performed RNA sequencing (RNA‐seq). A total of four samples, consisting of two wild‐type (WT) and two heterozygous mutant clones, were analyzed for differential expression gene (DEG) analysis. Each condition included two biological replicates, resulting in a total of four data sets for both the wild‐type and heterozygous conditions (Figure 6A). When genes with statistically significant 1.5‐fold or greater differences were extracted from the DEG list common to each genotype, 100 genes were upregulated and 88 genes were downregulated in SPTAN1^+^/^−^ organoids (Figure 6B). Then, Gene ontology (GO) analysis revealed that multiple calcium‐related and synaptic‐related terms, as well as cell adhesion processes were altered by SPTAN1 mutations (Figure 6C). This result is quite intriguing, considering that SPTAN1 is a known causative gene for severe epilepsy, whereas the transcriptomic data was obtained from nephron organoids. Indeed, many calcium‐related genes associated with these GO term were up‐ or downregulated by mutations in SPTAN1 (Figure 6D). Additionally, pathway analysis revealed that the MAPK pathway, including JUN, was significantly affected by SPTAN1 mutation (Figure 6E). This finding is consistent with previous reports showing RAC1 regulates the MAPK pathway [31, 32] and FOS [33].

We then performed live calcium imaging on nephron organoids cultured under flow conditions. To identify distal nephrons during live imaging, we used PNA lectin, which co‐localized with SLC8A1 in fixed immunostained samples (Figure 6F). Fluorescence intensity of Fluo‐4, calcium dye, was measured in PNA‐positive tubules, revealing a significant increase in intracellular calcium levels in SPTAN1^+^/^−^ organoids, compared to WT (Figure 6G,H). These findings suggest that the reduction of SPTAN1 in ARPKD leads to altered calcium signaling and elevated calcium levels in PNA‐positive tubules.

### 2.6 Restoring SPTAN1 via CRISPR Activation Alleviated Cystic Phenotypes in ARPKD Model

**FIGURE 7:**
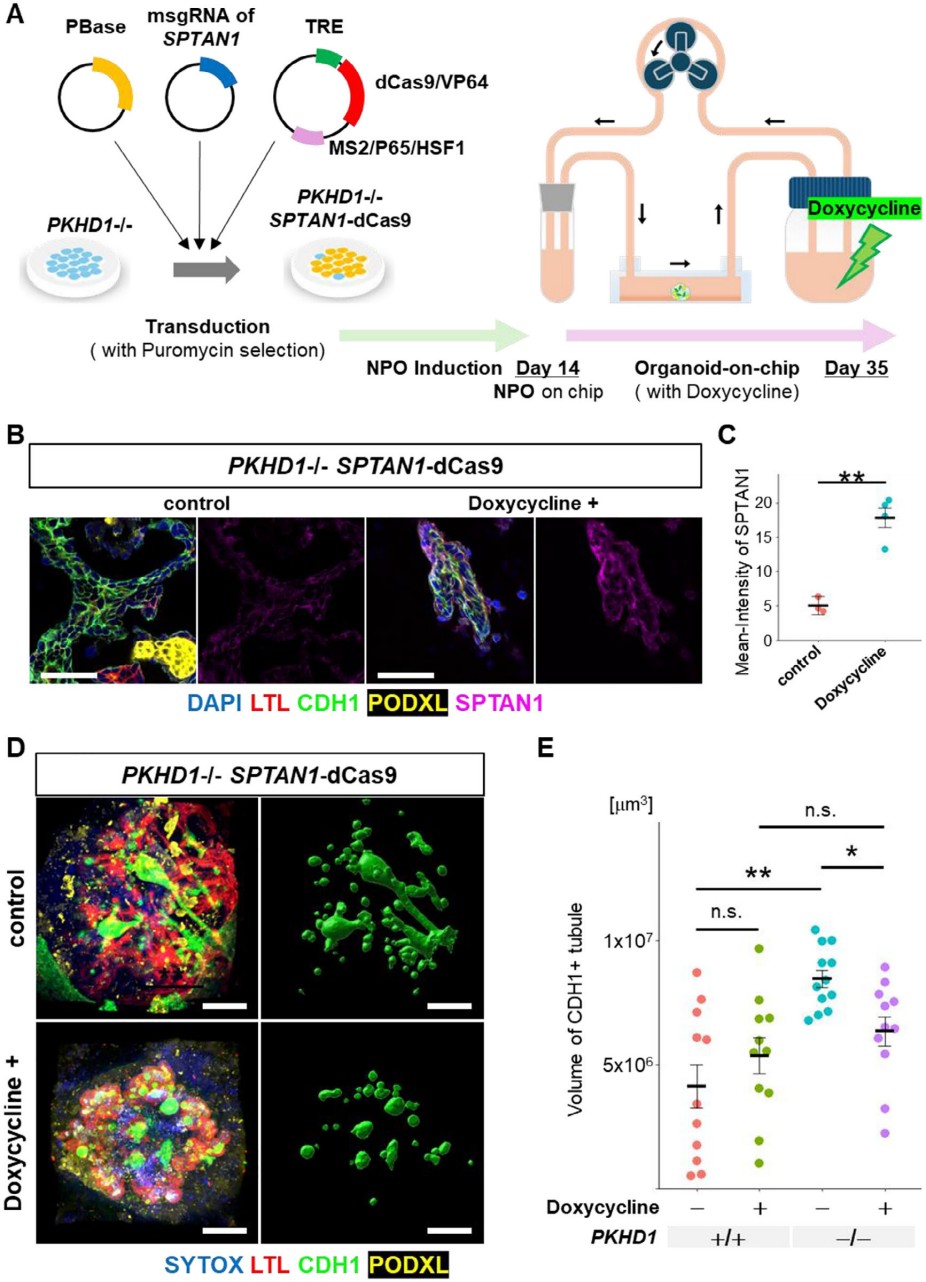
Restoring SPTAN1 in PKHD1^−^/^−^ nephron organoids. (A) Schematic representation of a protocol from transduction to flow culture in organoid‐on‐chip model. (B) Immunostaining for SPTAN1 in PKHD1^−^/^−^ SPTAN1‐dCas9 nephron organoids on day 35. Scale bars: 50 µm. (C) Quantification of the SPTAN1 intensity in CDH1+ tubules. Each dot represents the mean value of a single area. Each condition contains 3 areas selected randomly from plates. **p < 0.01, n.s.: not significant. (D) Left panels: Whole‐organoid 3D confocal imaging stacks of fluidic‐cultured PKHD1^−^/^−^ SPTAN1‐dCas9 organoids on day 35. Right panels: Imaris surface for CDH1. Scale bars: 200 µm. (E) Quantification of the total CDH1+ tubule volume in nephron organoids cultured under flow conditions. Each dot represents the value of a single organoid. Each condition contains 11–12 organoids. *p < 0.05. **p < 0.01, n.s.: not significant.

To determine whether reduced SPTAN1 expression is causative for ARPKD cyst formation, we tested whether restoring SPTAN1 in ARPKD organoids could alleviate disease phenotypes. To achieve this, we employed CRISPR activation, combining the tetracycline‐inducible dead Cas9 (dCas9) and Synergistic Activation Mediator (SAM) systems to upregulate SPTAN1 under the control of a tetracycline response element (TRE) [34, 35, 36]. To prevent silencing of the inserted dCas9 and SAM system sequences, we utilized PiggyBac transduction. We transfected PKHD1 homozygous ESCs with vectors encoding dCas9/VP64, a complex of additional activators (MS2/P65/HSF1), and four guide RNAs (gRNAs) targeting the SPTAN1 promoter region (Figure 7A). ESCs that survived puromycin selection were designated as PKHD1^−^/^−^ SPTAN1‐dCas9 lines. To validate the effect of SPTAN1 upregulation, we generated a negative control line (PKHD1^−^/^−^ dCas9) lacking SPTAN1 gRNAs but containing dCas9/VP64 and MS2/P65/HSF1. Since SPTAN1 is also expressed at the pluripotent stage, we examined its induction at this stage. Doxycycline administration led to increased SPTAN1 expression in PKHD1^−^/^−^ SPTAN1‐dCas9 cells but not in the PKHD1^−^/^−^ dCas9 control line (Figure S8A,B). We also confirmed doxycycline‐induced SPTAN1 upregulation in nephron organoids on day 35 of differentiation (Figure 7B,C).

Nephron organoids derived from the PKHD1^−^/^−^ SPTAN1‐dCas9 line were transferred onto a microfluidic chip on day 14 of differentiation and cultured under flow conditions for three weeks, with or without doxycycline. Immunostaining of nephron organoids on day 35 following fluidic culture revealed a significant reduction in the total volume of distal nephrons upon doxycycline treatment (Figure 7D,E). Additionally, SPTAN1 rescue by CRISPR activation significantly reduced Ki67+ proliferating cells in CDH1+ tubules in PKHD1^−^/^−^ organoids cultured under flow (Figure S8C,D). To evaluate potential off‐target effects of SPTAN1 activation, we generated an SPTAN1‐dCas9 line without PKHD1 mutations and cultured it under the same conditions. Doxycycline treatment did not significantly affect nephron differentiation, suggesting that CRISPR‐based activation of SPTAN1 may be a safe therapeutic approach for patients (Figure 7E). Similar findings were reproduced in iPSC‐derived PKHD1^−^/^−^ lines with SPTAN1‐dCas9 (Figure S9A,B).

**FIGURE 8:**
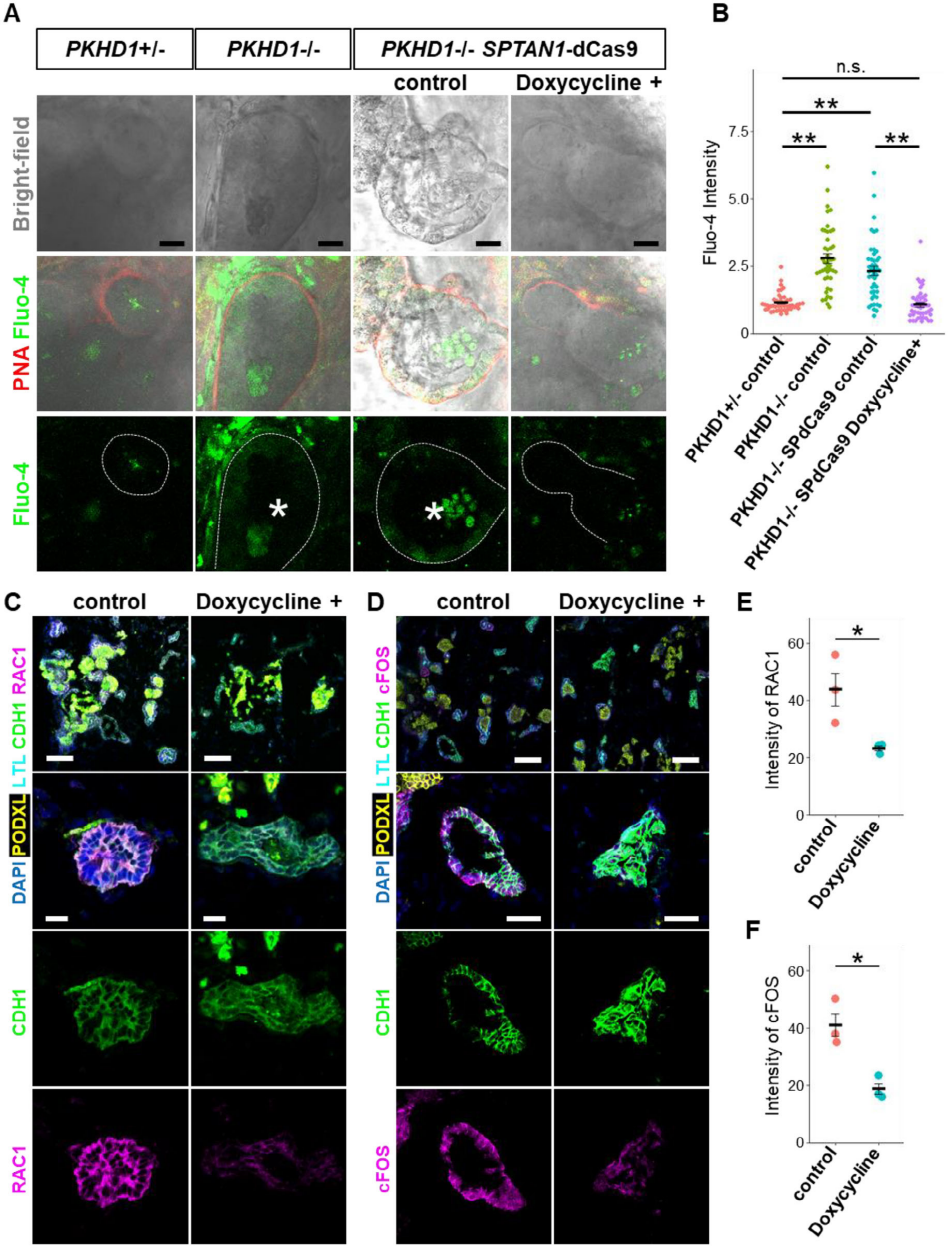
Molecular alterations by SPTAN1 supplementation. (A) Ca imaging by Fluo‐4 in fluidic‐cultured PKHD1^+^/^−^, PKHD1^−^/^−^, and PKHD1^−^/^−^ SPTAN1‐dCas9 organoids. Asterisks indicate dilated lumens. Scale bars: 20 µm. (B) Quantification of the Fluo‐4 intensity in PNA+ tubules. Each dot represents the mean value of a single epithelial cell. Each condition contains 45 epithelial cells from 3 organoids on day 35. **p < 0.01, n.s.: not significant. (C) Immunostaining for RAC1 in fluidic‐cultured PKHD1^−^/^−^ SPTAN1‐dCas9 organoids on day 35. Scale bars: upper panels, 100 µm; lower panels, 20 µm. (D) Immunostaining for cFOS in fluidic‐cultured PKHD1^−^/^−^ SPTAN1‐dCas9 organoids on day 35. Scale bars: upper panels, 100 µm; lower panels, 40 µm. (E) Quantification of the RAC1 intensity in CDH1+ tubules. Each dot represents the mean value of a single organoid. Each condition contains 20 and 14 tubules respectively from 3 organoids. *p < 0.05. (F) Quantification of the cFOS intensity in CDH1+ tubules. Each dot represents the mean value of a single organoid. Each condition contains 12 tubules from 3 organoids. *p < 0.05.

Lastly, we performed live calcium imaging and immunostaining to investigate the mechanistic effects of SPTAN1 activation in ARPKD. Compared to PKHD1^+^/^−^nephron organoids, PKHD1^−^/^−^ nephron organoids exhibited elevated intracellular calcium levels in the CNT epithelium. However, in PKHD1^−^/^−^ SPTAN1‐dCas9 organoids, doxycycline treatment reduced intracellular calcium levels to those observed in PKHD1^+^/^−^ controls (Figure 8A,B). Furthermore, CRISPR‐based SPTAN1 activation attenuated the upregulation of RAC1 and cFOS in PKHD1^−^/^−^ organoids (Figure 8C–F). These experiments were conducted in multiple batches, independent from the batch used for RNA‐seq, supporting the reproducibility of our findings regarding the role of SPTAN1. Our results suggest that restoring SPTAN1 alleviates cystic pathology while normalizing RAC1/cFOS activity and intracellular calcium levels in PKHD1^−^/^−^ organoids. Thus, our study demonstrates SPTAN1 as a key player in ARPKD cyst formation and a potential therapeutic target.

## 3 Discussion

ARPKD is a leading cause of renal failure and the need for kidney transplantation in children [1, 37]. Even today, no curative therapy has been established. While our previous study identified RAC1 and cFOS suppression as a potential therapeutic strategy for ARPKD [11], the role of these factors in cystogenesis remains an important question. In this study, we leveraged an organoid‐on‐chip model to replicate ARPKD pathogenesis and identified the RAC1/SPTAN1 pathway as a key regulator of cyst formation. Furthermore, we demonstrate that epigenetic upregulation of SPTAN1 mitigates cystic pathology, establishing SPTAN1 signaling as a potential therapeutic target for ARPKD. However, the organoid‐on‐chip model has certain limitations. First, because organoids are derived from stem cells, variations among clones must be considered. To minimize the effects of clonal variation, we used two clones each of WT and SPTAN1^+^/^−^ in our experiments. In addition, the reproducibility of the results was confirmed in both ESCs and iPSCs in the CRISPR activation of SPTAN1 experiment. We also minimized potential batch effects by analyzing different batches for microarray and immunostaining, RNA‐seq, and calcium imaging, all of which produced consistent results. Moreover, in the organoid‐on‐chip model, mechanical stimulation is applied to the organoids via medium flow, which includes two types: stretching stimulation from the basal side and tubular luminal flow stimulation. In the present organoid‐on‐chip model, we evaluated tubular dilation using an organoid‐level 3D volumetric metric to provide an objective and reproducible assessment across experiments. Using this approach, PKHD1^−^/^−^ organoids exhibited a ≥ 4‐fold increase in total CDH1‐positive distal tubule volume relative to controls, which is highly comparable to the magnitude observed in our prior organoid model. While our dextran perfusion experiments suggested the presence of tubular luminal flow, the luminal mechanical stress may be less compared to in vivo conditions. To address these limitations, we complemented our findings with in vivo analyses using transgenic mouse and patient samples.

SPTAN1 encodes the scaffolding protein α‐II‐spectrin, which is widely expressed across tissues [38]. In addition to its structural role, SPTAN1 regulates the stabilization of various cellular functions, including ion channels, cell adhesion molecules, and membrane transporters. It is also a well‐known causative gene in severe epilepsy and, together with actin, forms a submembrane cytoskeleton that provides mechanical elasticity to neuronal processes and mediates signal transduction [39]. In rodents, homozygous mutations in SPTAN1 are embryonic lethal, suggesting its crucial role during development, consistent with our results of failed organoid differentiation from SPTAN1^−^/^−^ [38]. However, to date, no studies have reported an association between SPTAN1 mutations and polycystic kidney disease. Our global gene expression analysis in flow‐cultured PKHD1^−^/^−^ organoids identified a decrease in SPTAN1, a potential RAC1 interaction partner, in cystic epithelia. SPTAN1 reduction was also validated in the cystic epithelium in ARPKD patients. Our SPTAN1^+^/^−^ organoids cultured under flow reproduced segment‐specificity of distal nephron cysts observed in ARPKD patients. Similar findings were observed in Sptan1^+^/^−^ mice. While previous ARPKD mouse models have been reported to recapitulate the proximal tubule‐derived cyst formation seen in the early to mid‐fetal period of ARPKD, Sptan1 mutant mice exhibit postnatal histological features resembling those of ARPKD, further supporting the role of SPTAN1 in cystogenesis [2, 25]. A clearer assessment of human–mouse similarity will require molecular profiling in human samples to define points of concordance with the Sptan1 model; additionally, engineering a double‐mutant Sptan1;Pkhd1‐/‐ line could further enhance human disease fidelity.

Although clinical reports are limited, there is a single case of a patient with a premature stop codon in SPTAN1 (NM_001130438.2; [2950C>T], NP_001123910.1: [R984X]) with prenatal renal cysts despite no family history of PKD [21]. This is consistent with a case report describing renal cysts associated with heterozygous nonsense mutations, likely resulting in a heterozygous knockout (het‐KO). This phenomenon was simulated in our nephron organoids carrying the nonsense mutation, which reproduced cystic pathology. Our study is the first to establish a causative relationship between PKHD1, SPTAN1, and distal‐dominant cyst formation using transgenic organoid and mouse models, as well as ARPKD patient samples. However, further research is needed to characterize mutation‐specific phenotypes, expand clinical case data, and strengthen in vivo validation, given that our findings in mice were limited by poor breeding efficiency, restricting our sample size. In future studies, crossing PCK rats with SPTAN1‐activated rats, which is not currently available, would provide valuable insight and further support our findings.

To validate our findings, we restored SPTAN1 expression and observed an alleviation of cystic phenotypes in PKHD1^−^/^−^ nephron organoids. Notably, both distal nephron dilation and RAC1/cFOS upregulation were suppressed, indicating that SPTAN1 is critical for the RAC1–cFOS upregulation associated with this phenotype. Furthermore, treatment with a RAC1 inhibitor mitigated the cystic phenotype in SPTAN1 mutant organoids, consistent with the view that SPTAN1 restrains RAC1 activation to maintain tubular morphology [18]. Mechanistically, we propose that SPTAN1, as a principal cortical scaffold, spatially regulates the access of cytosolic RAC1 regulators (GEFs/GAPs) to submembrane microdomains; SPTAN1 reduction would loosen this lattice, increase local GEF access, and thereby elevate RAC1 activity. Given that mechanical input can drive RAC1–FOS signaling and that FOS promotes cell proliferation [33, 40, 41], heightened RAC1–FOS activity provides a plausible route to aberrant proliferation and cyst formation in ARPKD.

Additionally, epithelial calcium levels were altered in distal nephrons of SPTAN1^+^/^−^ organoids, indicating an involvement of SPTAN1 in calcium signaling regulation. Dysregulated calcium signaling has long been implicated in cyst formation in PKD, it has been reported that calcium signaling is reduced in PCK rats, and that increasing calcium levels can suppress cyst formation [42]; however, the underlying mechanisms remain incompletely understood [43, 44]. Given that mechanical stimulation via primary cilia influences calcium signaling [45, 46], our analysis using flow culture in a mechanically stimulated environment may offer novel insights that were previously unexplored. However, the present study did not elucidate the molecular mechanism by which PKHD1 mutations lead to the downregulation of SPTAN1. Previous studies have reported that the C‐terminal fragment of cleaved fibrocystin, the protein encoded by PKHD1, translocate into the nucleus and modulates transcriptional activity [47, 48, 49]. It is therefore conceivable that SPTAN1 represents one of its transcriptional targets. Future studies employing approaches such as ChIP‐sequencing targeting the C‐terminal domain of PKHD1 will be required to delineate this regulatory mechanism.

This study identifies SPTAN1 as a key regulator in ARPKD cystogenesis and highlights it as a potential therapeutic target for ARPKD. With the advent of efficient gene editing technologies such as CRISPR, therapeutic applications of gene correction for inherited diseases are emerging. However, reliance on endogenous DNA repair mechanisms makes it challenging to achieve a single desired change due to the complexity of these pathways [50]. This includes unexpected mutations in non‐target genes (off‐target effects) and the necessity of introducing silent mutations in target genes. An alternative approach to regulating gene function has recently gained significant interest: rewriting the epigenetic landscape to control gene expression without altering the underlying DNA sequence. This epigenome editing strategy, which combines CRISPR technology with VP64, a transcriptional activator, and TET1, which induces DNA demethylation, has emerged as a promising therapeutic option [51]. It has already begun to be applied in clinical settings, including for hereditary diseases [52]. Our findings suggest that epigenomic activation of SPTAN1 expression may serve as a fundamental treatment strategy for ARPKD patients. Furthermore, the absence of apparent adverse effects by SPTAN1 regulation in wild‐type is a promising factor for clinical application. Although challenges remain, selective kidney‐targeted treatment is under investigation [53]. Future studies, particularly those addressing delivery methods, are expected to pave the way for epigenomic SPTAN1 modulation as a novel therapeutic option for ARPKD patients.

This study also investigated the origin of ARPKD cysts. Traditionally, ARPKD cysts have been thought to originate primarily from the collecting ducts, based on the morphological diagnosis of dilated cystic epithelium [54]. Intriguingly, in the scRNA‐seq of KPMP, PKHD1 is expressed in CCD, but not in IMCD (https://atlas.kpmp.org/explorer/dataviz). Our scRNA‐seq primarily included the CCD, but not the IMCD, for collecting duct assessment, as all 24 datasets were obtained from percutaneous renal biopsies, which typically lack medullary tissues. However, our scRNA‐seq analysis of healthy kidneys revealed that PKHD1 is highly expressed not only in the collecting ducts but also in the connecting tubules. Furthermore, we identified SLC8A1 as a potential marker to distinguish distal convoluted tubules/connecting tubules from collecting ducts and found that cystic epithelia from ARPKD patients expressed SLC8A1, suggesting that ARPKD cysts may also originate from not only from the collecting ducts but also from the distal convoluted tubules/connecting tubules. Supporting this hypothesis, we observed selective dilation of SLC8A1‐positive tubules in both PKHD1^−^/^−^ and SPTAN1^+^/^−^ nephron organoids. Consistent results were also obtained in Sptan1 mutant mice. Meanwhile, there was no significant difference in SLC8A1‐positive tubules between wild‐type nephron organoids cultured with or without flow. Additionally, the proportion of SLC8A1‐positive tubules increased under flow conditions, suggesting that mechanical stimulation from fluidic culture promotes connecting tubule differentiation and maturation. However, given the possibility of secondary enhanced expression of SLC8A1 in the cyst epithelium, we cannot exclude the possibility that some renal cysts may originate from the collecting duct (CD), even though most cysts in ARPKD patients were SLC8A1‐positive in the analyzed cases. Conversely, it is also possible that cysts lose SLC8A1 expression, and we indeed observed minor cystic populations lacking SLC8A1. To precisely determine the origin of ARPKD cysts, lineage tracing using Sptan1 mutant mice and/or organoid models through selective directed differentiation may be beneficial. Further studies are needed to confirm these findings.

## 4 Conclusions

**FIGURE 9:**
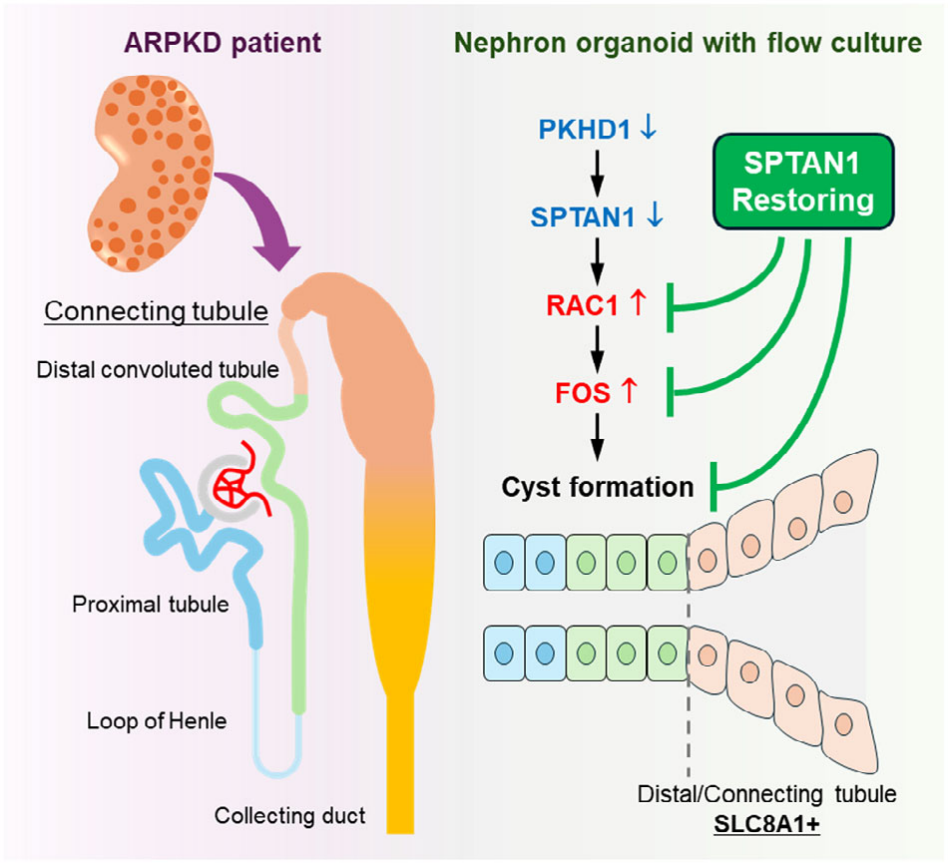
Distal/connecting tubules expressing SLC8A1 have been suggested as a potential origin of ARPKD cysts. SPTAN1 has been identified as a key molecule in ARPKD cyst formation. Restoring SPTAN1 in PKHD1^−^/^−^ organoids reduced cyst formation and decreased RAC1/c‐FOS expression, highlighting SPTAN1's role in ARPKD and the potential of epigenome editing as a therapeutic approach.

Through a multifaceted approach using organoid‐on‐chip models, transgenic mice, and patient kidney samples, we identified SPTAN1 as a key regulator of cystic pathology in ARPKD. SPTAN1‐mutant organoids exhibited distal‐nephron cysts, increased RAC1/c‐FOS expression, and elevated intracellular calcium. Single‐cell RNA sequencing revealed SLC8A1 as a marker distinguishing distal/connecting tubules from collecting ducts, with SLC8A1 expression detected in cystic epithelia of ARPKD patients, suggesting that ARPKD cysts originate from the distal/connecting tubules. Notably, restoring SPTAN1 in PKHD1^−^/^−^ organoids alleviated cystic phenotypes, normalized intracellular calcium, and reduced RAC1/c‐FOS expression (Figure 9). These findings establish SPTAN1 as a central regulator of ARPKD pathogenesis and highlight epigenome editing as a potential therapeutic strategy, paving the way for the translation of findings from basic research into clinical practice.

## 5 Experimental Section

### 5.1 Cell Culture and Maintenance

H9 (WiCell Research Institute, WA09) human ES cells, H9‐derived PKHD1 mutants, H9‐derived SPTAN1 mutants, and H9‐derived PKHD1mutant with SPTAN1‐dCas9 line were maintained on Vitronectin (Thermo Fisher Scientific, A14700)–coated plates using StemFit Basic04 Complete Type (Ajinomoto Co. Inc.), in accordance with previously reported methods [5].

### 5.2 Nephron Organoid Differentiation

Nephron organoids were differentiated and maintained following previously established protocols [4, 5]. Quality check was performed by immunostaining for SIX2 on day 8 and for LTL, CDH1, and PODXL, segment‐specific markers of nephron on day 21. For static culture, the basal medium consisting of Advanced RPMI (ARPMI; Thermo Fisher Scientific, 12633‐012) and GlutaMAX (Thermo Fisher Scientific, 35050–061) was replaced every 2 to 3 days.

### 5.3 Generation of Heterozygous SPTAN1‐Mutant hPSCs and H9‐Derived PKHD1mutant With SPTAN1 Enhancement Line

The PX459 plasmid (Genscript), encoding enhanced *Streptococcus pyogenes* Cas9 and single‐guide RNA (sgRNA) targeting the first exon of SPTAN1 (5′‐AGACCGATACCACCGCTTCA‐3′) was designed to generate heterozygous and homozygous SPTAN1 mutant lines. On the passage day, 15 000 hESCs were seeded into one well of a six‐well plate. On day 5 after passage, 1.5 µg of the PX459 plasmid was mixed with 6 µl of the Lipofectamine Stem Transfection Reagent (Thermo Fisher Scientific, STEM00001) and transfected into the cells in a six‐well plate. After 24 h of lipofection, transfected cells were selected using transient puromycin treatment (1 µg mL^−1^ for 24 h). Surviving cells were cultured in StemFit medium with ROCK inhibitor for three days and then dissociated with Accutase for single‐cell passage. Individual clones were expanded from single cells. CRISPR‐Cas9–induced mutations in individual clones were identified via deep sequencing (Genome Technology Access Center, McDonnell Genome Institute, MO). The clone without SPTAN1 mutations among those generated was used as an isogenic control for subsequent experiments.

To enhance SPTAN1 expression in PKHD1 mutant line, we employed the CRISPR activation system in combination with the Synergistic Activation Mediator (SAM) system [34, 35, 36]. Additionally, we utilized the PiggyBac system to avoid genomic silencing concerns [55]. A vector containing the Tetracycline‐responsive element promoter/dCas9/VP64 and MS2/P65/HSF1 (dCas9 vector), gRNAs vector including four guide sequences (5′‐ ACGCGCGCTCCCGCGGCGGG ‐3′, 5′‐ CCCCACCCCTCGCGCAGCGC ‐3′, 5′‐ CGGTCGGGCGGCGAGCCGGC ‐3′, 5′‐ AGGAGCCGAAGCGACAATGC ‐3′) and the PBase vector (VectorBuilder), were designed. On the passage day, 15 000 hESCs or hiPSCs were seeded into one well of a six‐well plate. Based on previous reports, 1 µg of dCas9 vector, 1 µg of gRNAs vector and 10 µg of PBase vector, was mixed with 6 µl of the Lipofectamine Stem Transfection Reagent and transfected into the cells in a six‐well plate on day 5 after passage [56]. After 48 h of lipofection, transfected cells were selected using transient puromycin treatment (0.5 µg mL^−1^ for 5 days). After two days culture in StemFit medium with ROCK inhibitor, surviving cells were stocked as PKHD1^−^/^−^ SPTAN1‐dCas9 line. Simultaneously, same transfection excluding gRNAs vector was performed to establish PKHD1^−^/^−^ dCas9 line and PKHD1^+^/^+^ dCas9 line as control lines.

### 5.4 Culturing Kidney Organoid‐on‐Chip With Medium Flow

On day 14 of differentiation, the 4–6 organoids were transferred into a microfluidic chip (ibidi, 10841) loaded with 300 µl medium composed of ARPMI with GlutaMAX and 10% Geltrex (Thermo Fisher Scientific, A1413302). The chips were incubated overnight in an incubator. On day 15, the microfluidic chip was connected to a flow system (Tokaihit, BPU, Japan) prewarmed with 36 mL medium in the reservoir. When connecting multiple chips in series, a connecting tube was plugged from the outlet of the chip to the inlet of the next chip. After connecting all chips to flow system, the organoid‐on‐chip were cultured up to day 35 in pulsatile flow mode, with flow rates alternating between 0.4 and 0.04 mL min^−1^ at 0.5 s intervals. Half of the medium in the reservoir, ARPMI with GlutaMAX, was replaced every 2 to 3 days. For RAC1 inhibition, NSC23766 (Abcam, ab142161) 20 µM was added to ARPMI from day15. For restoring SPTAN1, doxycycline 1 µg mL^−1^ was added to ARPMI from day 15. In our microfluidic setup, the trans‐chip pressure differential was estimated at ∼5–20 mmHg, below adult intraglomerular pressure (∼50–60 mmHg), but within a range compatible with developmental mechanosensory stimuli.

### 5.5 Immunocytochemistry of Kidney Organoids

Nephron organoids were fixed with 4% paraformaldehyde (PFA) for 1 h at RT, incubated in 30% sucrose overnight at 4°C, and embedded in optimal cutting temperature (OCT) compound, and 10‐µm frozen sections were cut using a cryostat. Samples were blocked with blocking buffer (BB; 5 wt.% donkey serum in PBS containing 0.3 wt.% Triton X‐100) for 1 h at RT and then incubated with primary antibodies in antibody dilution buffer [ADB; 1% bovine serum albumin (BSA) containing 0.3% Triton X‐100] over night at 4°C. The following antibodies and lectins were used in these studies: anti‐CDH1 (1:200; Abcam, ab11512), LTL (1:200; Vector Laboratories, B‐1325), anti‐PODXL (1:500; R&D Systems, AF1658), anti‐ TUBA1A (1:200; Abcam, ab179484), anti‐ATP1A1 (1:100; DSHB, A5‐DSHB), anti‐SPTAN1 (1:100; Invitrogen, MA1‐91103), anti‐RAC1 (1:100; Developmental Studies Hybridoma Bank, CPTC RAC1‐2), anti‐FOS (1:500; Abcam, ab190289), anti‐SLC8A1 (1:100; Invitrogen, MA3‐926), and PNA‐Cy5 (1:200; Vector Laboratories, CL‐1075‐1). For immunostaining with anti‐RAC1 and anti‐SLC8A1, samples were incubated with IHC Antigen Retrieval Solution (Invitrogen, 00‐4955‐58) for 2 min at 90°C before blocking with BB. For immunostaining with biotinylated LTL, the Streptavidin/Biotin Blocking Kit (Vector Laboratories, #SP‐2002) was used according to the manufacturer's protocol. After washing three times in PBS, samples were incubated with Alexa Fluor secondary antibodies in ADB for 2 h at RT. Vectashield with DAPI (Vector Laboratories) was added to stain the nucleus. Images were taken using a Leica STELLARIS 8 confocal microscope.

### 5.6 Quantification of Immunofluorescence Intensity

For quantification of RAC1 and cFOS immunofluorescence, we employed an intensity‐based approach rather than binary “positive/negative” cell scoring, because antigen retrieval and multicolor immunofluorescence resulted in elevated background signal that made it difficult to define a robust and reproducible intensity threshold. Therefore, fluorescence intensity–based quantification was used to capture relative differences in expression under identical staining and imaging conditions. Quantitative analysis was performed on CDH1‐positive tubular epithelial regions, which were manually selected based on single‐channel CDH1 images. Tubules showing clear LTL co‐positivity were excluded from the analysis. For each selected tubule, the tubular epithelial area was defined as the CDH1‐positive region excluding the lumen, and the mean fluorescence intensity of RAC1 or cFOS within this area was measured using ImageJ/Fiji. To allow comparison between tubules of different diameters, including normal and dilated tubules, fluorescence intensity was normalized to the tubular epithelial area. Background fluorescence was measured within non‐tissue regions of each section and subtracted on a per‐slide basis to minimize inter‐section and inter‐batch variability.″

### 5.7 Whole‐Mount Immunohistochemistry With Sample Clearing

The staining and clearing protocol were described previously [57]. The nephron organoids were fixed overnight by 4% PFA at 4°C. The microfluidic chips were also fixed directly with 4% PFA overnight at 4°C and stained. The following antibodies were used in these studies: anti‐CDH1 (1:200; Abcam, ab11512), LTL (1:200; Vector Laboratories, B‐1325), anti‐PODXL (1:500; R&D Systems, AF1658). Samples were examined with a Leica STELLARIS 8 confocal microscope and by using Imaris 3D software (Bitplane). Then, fully automated 3D imaging by a machine‐learning was applied for quantification of nephron segment‐volume, as described previously [58].

### 5.8 Human Subjects and Ethical Approval

Human ARPKD kidney tissues consisted of two postmortem cases: a 2‐month‐old female infant born at 35 weeks of gestation and a 7‐day‐old male infant born at 32 weeks of gestation. Kidney tissue samples were obtained from the archival pathology repository of Cedars‐Sinai Medical Center and were analyzed in a fully de‐identified manner. All specimens had been originally collected and processed as part of routine clinical care and subsequently archived. The use of archival human tissue for research was approved by the Institutional Review Board (IRB) of Cedars‐Sinai Medical Center (approval number: STUDY00001229). The IRB determined that the study met criteria for research on existing de‐identified specimens and therefore waived the requirement for informed consent in accordance with institutional policies and applicable regulations (including HIPAA privacy regulations). All procedures involving human material were performed in accordance with IRB approval and consistent with the principles of the Declaration of Helsinki.

### 5.9 Immunostaining of Human Tissue

Immunohistochemistry of human ARPKD samples and normal portions of tumor nephrectomy were carried out as described previously [11]. Briefly, 4‐µm‐thick paraffin sections were blocked with 3% H2O2 and 1% BSA–PBS, after deparaffinization, hydration, and antigen retrieval in 0.01 M citrate buffer (pH 6.0) at 95°C for 30 min. The sections were incubated overnight at 4°C with primary antibodies: anti‐human SPTAN1 antibody (Invitrogen, MA1‐91103) or anti‐SLC8A1 antibody (Invitrogen, MA3‐926). After washing with PBS three times, secondary reagents (HRP‐Goat anti‐Mouse IgM Antibody, Invitrogen) were applied, incubated at RT for 1 h, and visualized with ImmPACT DAB (Vector Laboratories). For immunofluorescence staining, 4‐µm‐thick paraffin sections from ARPKD samples and normal portions of tumor nephrectomy were blocked with 1% BSA in PBS for 1 h, after deparaffinization, hydration, and antigen retrieval in 0.01 M citrate buffer (pH 6.0) at 95°C for 30 min. The sections were incubated overnight at 4°C with following primary antibodies/reagent: SLC8A1 (1:25; MA3‐926, Invitrogen), ATP1A1 (1:50; A5‐DSHB, DSHB), and FITC‐conjugated LTL (1:100; FL‐1321‐2, Vector). After washing in PBS, Alexa fluor 555‐labeled goat anti‐mouse IgM (1:500; A‐21426, ThermoFisher) and Alexa fluor 488‐labeled donkey anti‐mouse IgG (1:500; A‐21202, ThermoFisher) were applied to detect SLC8A1 and ATP1A1, respectively. For FITC‐LTL staining, both incubation and washing buffers contained 1 mM CaCl_2_, and the conjugate was directly visualized. All procedures were conducted under a protocol approved by the Cedars‐Sinai Medical Center Institutional Review Board (STUDY00001229).

### 5.10 Animal Studies

All animal experiments were approved by the Mass General Brigham Institutional Animal Care and Use Committee (IACUC) (protocol number: 2023N000120) and conducted in accordance with institutional guidelines. Sptan1 mutant mice (C57BL/6NCrl‐Sptan1em1(IMPC)Mbp/Mmucd) were obtained from the UC Davis Mouse Biology Program (Mutant Mouse Resource & Research Center, MMRRC) and maintained on a C57BL/6N background under specific pathogen‐free conditions with a 12 h light/dark cycle and ad libitum access to food and water. Both male and female mice were used. Independent cohorts were analyzed at 16 weeks and 13 months of age, with MRI performed at 12 months in the aged cohort. For MRI analysis, mice were imaged in vivo at 12 months of age under isoflurane anesthesia (1%–3% in oxygen). Body temperature was maintained at 37°C using a rodent warming system, and respiration was monitored during imaging. For tissue collection, mice were euthanized by CO_2_ inhalation followed by cervical dislocation at 13 months of age. Kidneys were immediately harvested and measured. Separate kidney samples were either fixed in 4% paraformaldehyde at 4°C overnight for histological processing or homogenized in RIPA buffer for protein analyses. Fixed tissues were paraffin‐embedded (sectioned at 5 µm) or cryo‐embedded in OCT compound (sectioned at 10 µm). Sections were used for immunofluorescence analyses.

### 5.11 Magnetic Resonance Imaging (MRI) of Mice

MRI was performed on a 4.7T Bruker pharmascan magnet with T2 (TE: 40 ms, TR: 3000 ms, Matrix 256 × 256 × 16, Voxel size (0.15625 × 0.15625 × 0.7 mm)) and a similar T2 scan with chemical fat saturation via a hermitian spoiler pulse approximately 3.5 ppm down from the water peak. Kidney and lesion volumes were analyzed manually using Amira (version 5.4, Thermoscientific) and scans were visualized using Horos (https://horosproject.org/).

### 5.12 Immunofluorescence on Mouse Kidney Sections

Paraffin sections of 16‐week old mouse kidneys were obtained from IMPC, and 13‐month‐old kidneys were collected in‐house as described in the Animal studies section. Paraffin sections were deparaffinized in xylene and rehydrated through graded ethanol. Both paraffin and cryosections were subjected to antigen retrieval using IHC Antigen Retrieval Solution (Invitrogen, 00‐4955‐58) for 20 min at 90°C. Samples were blocked with BB for 1 h at RT and then incubated with primary antibodies in ADB over night at 4°C. The following antibodies were used in these studies: anti‐CDH1 (1:200; Abcam, ab11512), anti‐SLC8A1 (1:100; Invitrogen, MA3‐926), LTL (1:200; Vector Laboratories, B‐1325). For immunostaining with biotinylated LTL, the Streptavidin/Biotin Blocking Kit (Vector Laboratories, #SP‐2002) was used according to the manufacturer's protocol. After washing three times in PBS, samples were incubated with Alexa Fluor secondary antibodies in ADB for 2 h at RT. Vectashield with DAPI (Vector Laboratories) was added to stain the nucleus. Images were taken using a Leica STELLARIS 8 confocal microscope.

### 5.13 Rac1 Activity Assay

Rac1 activity was measured using the Rac1 G‐LISA GTPase Activation Assay Kit (Cytoskeleton, Inc., BK128). All samples were obtained from the kidney cortex. The tissues were processed in RIPA buffer supplemented with protease inhibitors, homogenized using a sonicator, and the resulting homogenates were stored at −80°C until analysis. Before the assay, samples were thawed on ice and centrifuged to obtain clarified lysates. Protein concentrations were then measured, and all lysates were normalized to 0.8 mg mL^−1^, which falls within the manufacturer‐recommended range of 0.25–1.0 mg mL^−1^ for the assay. The G‐LISA assay was performed according to the manufacturer's instructions. Equal amounts of normalized lysates were loaded into the wells, and Rac1‐GTP levels were quantified by measuring absorbance using a microplate reader. Absorbance values were corrected by subtracting blank readings prior to analysis.

### 5.14 Dextran Perfusion Assay

On day 21, nephron organoids cultured on a microfluidic chip were treated with WGA‐conjugated 647 (5 µg mL^−1^; Thermo Fisher Scientific, W32466), as described previously [59]. After incubation at 37°C for 1 h under flow, LMD (10 µg mL^−1^; Thermo Fisher Scientific, D3306) was added to a medium reservoir. At this time, to reduce the total medium volume, we removed an original medium reservoir and utilized the pressure buffer tube as a medium reservoir, thereby reducing the total medium volume to 5 mL. Samples were examined in the fluidic condition using a Leica STELLARIS 8 confocal microscope. In static condition, LMD (10 µg mL^−1^) was directly added to the chip. Static samples were examined without medium flow using a Leica STELLARIS 8 confocal microscope. For the comparison of dextran intensity, regions of interest (ROIs) were placed within the lumens of five WGA‐positive tubules under both flow and static conditions, and dextran intensity was measured over time. Statistical evaluation was performed using a linear mixed‐effects model.

### 5.15 DNA Microarray and RNAseq

The DNA microarray reanalysis of PKHD1 mutant kidney organoids utilized data previously deposited in the Gene Expression Omnibus (www.ncbi.nlm.nih.gov/geo/) under accession number GSE190272. A heatmap was created using R version 4.4.1 (R Foundation for Statistical Computing, Vienna, Austria; http://www.R‐project.org/). Total RNA was isolated from flow‐cultured kidney organoids using TRIzol (Thermo Fisher) from two biological replicates per condition (SPTAN1^+^/^+^ clone 1, SPTAN1^+^/^+^ clone 2, SPTAN1^+^/^−^ clone 1, SPTAN1^+^/^−^ clone 2) for RNA‐Seq analysis of the SPTAN1 mutation. The RNA‐Seq data was generated using the NovaSeq X Plus (Novogene), and all analyses were performed using NovoMagic, a free data analysis platform provided by Novogene. The analysed data are included in the Supporting files.

### 5.16 Single‐Cell RNA‐Seq

Uniform Manifold Approximation and Projection (UMAP) was performed using the following 24 participants from KPMP; 163‐2, 163‐3, 163–4, 163–5, 163–7, 164‐10, 164‐13, 164‐15, 164‐19, 164‐20, 164‐22, 164‐3, 164–6, 165‐14, 165–9, PRE018‐1, PRE019‐4, PRE027, PRE038, PRE055‐1, PRE062‐1, PRE19‐025, PRE19‐05, PRE98sc. Abbreviations in UMAP are as follows: Po, podocytes; PT, proximal tubules; LoH, loop of Henle; DCT, distal convoluted tubules; CNT, connecting tubules; CD‐PC, collecting duct principal cells; IC, intercalated cells; FB, fibroblast cells; EC, endothelial cells; VSM, vascular smooth muscle cells; ImC, immune cells. UMAP and a violin plot were created using Seurat version 5.2.0 [60] and R version 4.4.1 (R Foundation for Statistical Computing, Vienna, Austria; http://www.R‐project.org/). The codes were described in the Supporting file.

### 5.17 Calcium Imaging of Nephron Organoids

After one week of flow culture from day 14, PNA‐Cy5 (1:200; Vector Laboratories, CL‐1075‐1) and Fluo‐4 (1:1000; Life Technologies, F10489) were introduced into the medium containing nephron organoids on chips. At this stage, the loading buffer was also added according to the manufacturer's manual of Fluo‐4. Following incubation at 37°C for 60 min under medium flow, Fluo‐4 fluorescence was observed directly on the chip using a Leica STELLARIS 8 confocal microscope. To specifically evaluate intracellular Ca levels in tubular epithelial cells, quantitative analyses were restricted to PNA‐positive epithelial regions. Regions of interest (ROIs) were manually defined based on single‐channel PNA images and excluded luminal areas and surrounding non‐epithelial tissue. For each selected tubule, mean Fluo‐4 fluorescence intensity was measured within the PNA‐positive epithelial area. To allow comparison between tubules of different sizes, fluorescence intensity was normalized to the area of PNA‐positive epithelial cells. Background fluorescence was measured within non‐tissue regions of each field and subtracted prior to analysis.

### 5.18 Statistical Analysis

Bars in all data indicate mean ± standard error (SE). Statistical analysis was done by ANOVA; Tukey's multiple comparison test using PMCMRplus (Calculate Pairwise Multiple Comparisons of Mean Rank Sums Extended. R package version 1.9.12; https://CRAN.R‐project.org/package = PMCMRplus) and R version 4.4.1 (R Foundation for Statistical Computing, Vienna, Austria; http://www.R‐project.org/), and statistical significance was attributed to values of *p* < 0.05 as determined by Tukey's multiple comparison test. Different significance levels (*p* values) were indicated in each figure with asterisks: *p < 0.05, **p < 0.01, ***p < 0.001, and ****p < 0.0001.

For transparency, we state here the number of times experiments were repeated independently, with similar results obtained, to produce the data shown. Figure 1C: Two independent experiments were performed for fluidic culture, and single experiment was performed for static culture. Control included PKHD1^+^/^+^ and PKHD1^+^/^−^ nephron organoids. Figure 2F: Single experiment was performed. Each condition contains 3 organoids. Figure 2H: One healthy control tissue and one ARPKD patient tissue were analyzed. Figure 3B,C: Three independent experiments were performed. Each experiment was performed with two SPTAN1^+^/^+^ clones and SPTAN1^+^/^−^ clones. Figure 3E,G,I: Two independent experiments were performed. Figure 4C: Four wild type mice and five Sptan1^+^/^−^ mice were analyzed. Figure 4E: Two male kidneys and two female kidneys were analyzed for each genotype. Figure 5D: Two independent experiments were performed. Total 249 tubules were analyzed. Figure 5F: Two independent experiment was performed. Each condition contains 30 and 18 tubules respectively. Figure 5H: One healthy control tissue and two ARPKD patient tissues were analyzed. Figure 6H: Single experiment was performed. Each condition contains 45 tubular cells from three organoids. Figure 7C: Single experiment was performed. Figure 7E: Three independent experiments were performed. Figure 8B: Two independent experiments were performed. Each condition contains 45 epithelial cells from three organoids. Figure 8E,F: Single experiment was performed. Figure S1: Two independent experiments were performed for fluidic culture, and single experiment was performed for static culture. Figure S3B,C: Three independent experiments were performed. Figure S4A: four male kidneys and two female kidneys for wild type, six male kidneys and four female kidneys for Sptan1^+^/^−^ were analyzed. Figure S4B: Two male kidneys and two female kidneys were analyzed for each genotype. Figure S6A: Two independent experiments were performed. Figure S7B: Three male kidneys were analyzed for each genotype. Figure S8B: Single experiment was performed. Figure S8D: Single experiment was performed. Figure S9B: Single experiment was performed.

## Author Contributions

Methodology: **S.K**., **Y.H**., and **R.M**. Investigation: **S.K**., **Y.H**., **S.S**., **S.P**., **H.H**., and **S.I**. Visualization: **S.K**., **Y.H**. and **R.M**. Formal analysis: **S.K**. and **Y.H**. Data curation: **S.K**. Resources: **R.M**. Funding acquisition: **R.M**. Project administration: **M.Y**. and **R.M**. Supervision: **P.C.H**., **L.M.S**., **M.Y**., and **R.M**. Conceptualization: **R.M**. Writing – original draft: **S.K**. and **R.M**.

## Conflicts of Interest

R.M. is an inventor on a patent related to this work filed by President and Fellows of Harvard College and Mass General Brigham (PCT/ US2018/036677, filed on 8 June 2018, published on 13 December 2018). R.M. served as a scientific advisory member in Trestle Biotherapeutics. The authors declare no other competing interests.

## Supporting information




**Supporting File 1**: advs74532‐sup‐0001‐FigureS1‐S9.pdf.

**Supporting File 2**: advs74532‐sup‐0002‐DataSet.docx.

**Supporting File 3**: advs74532‐sup‐0003‐DataSet.xlsx.

**Supporting File 4**: advs74532‐sup‐0004‐DataSet.xlsx.

**Supporting File 5**: advs74532‐sup‐0005‐DataSet.xlsx.

## Data Availability

The data that support the findings of this study are openly available in Gene Expression Omnibus at www.ncbi.nlm.nih.gov/geo/, reference number 288738.
