# Supplementary material for: Deciphering the Impact of RAC1‐SPTAN1 in ARPKD Cystogenesis Using Multifaceted Models
Source: Adv Sci (Weinh). 2026 Feb 26;13(25):e24001. doi: 10.1002/advs.202524001 (PMC13137809; doi:10.1002/advs.202524001)
Supplement: Supplementary file 1 — Supporting File 1: advs74532‐sup‐0001‐FigureS1‐S9.pdf. [file ADVS-13-e24001-s002.pdf]

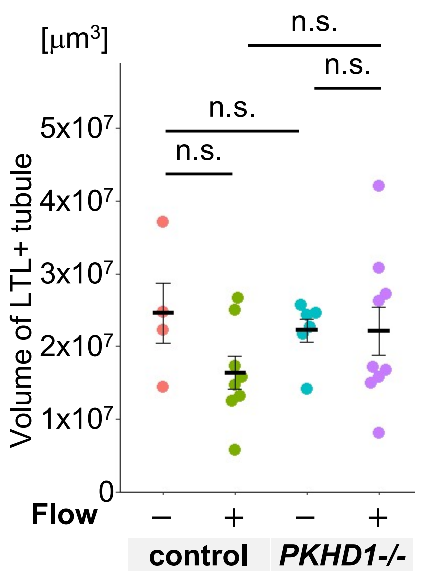

Figure S1: Quantification of the total LTL+ tubule volume in nephron organoids on day 35. Each dot represents the value of a single organoid. Each condition contains 5-9 organoids. n.s.: not significant.

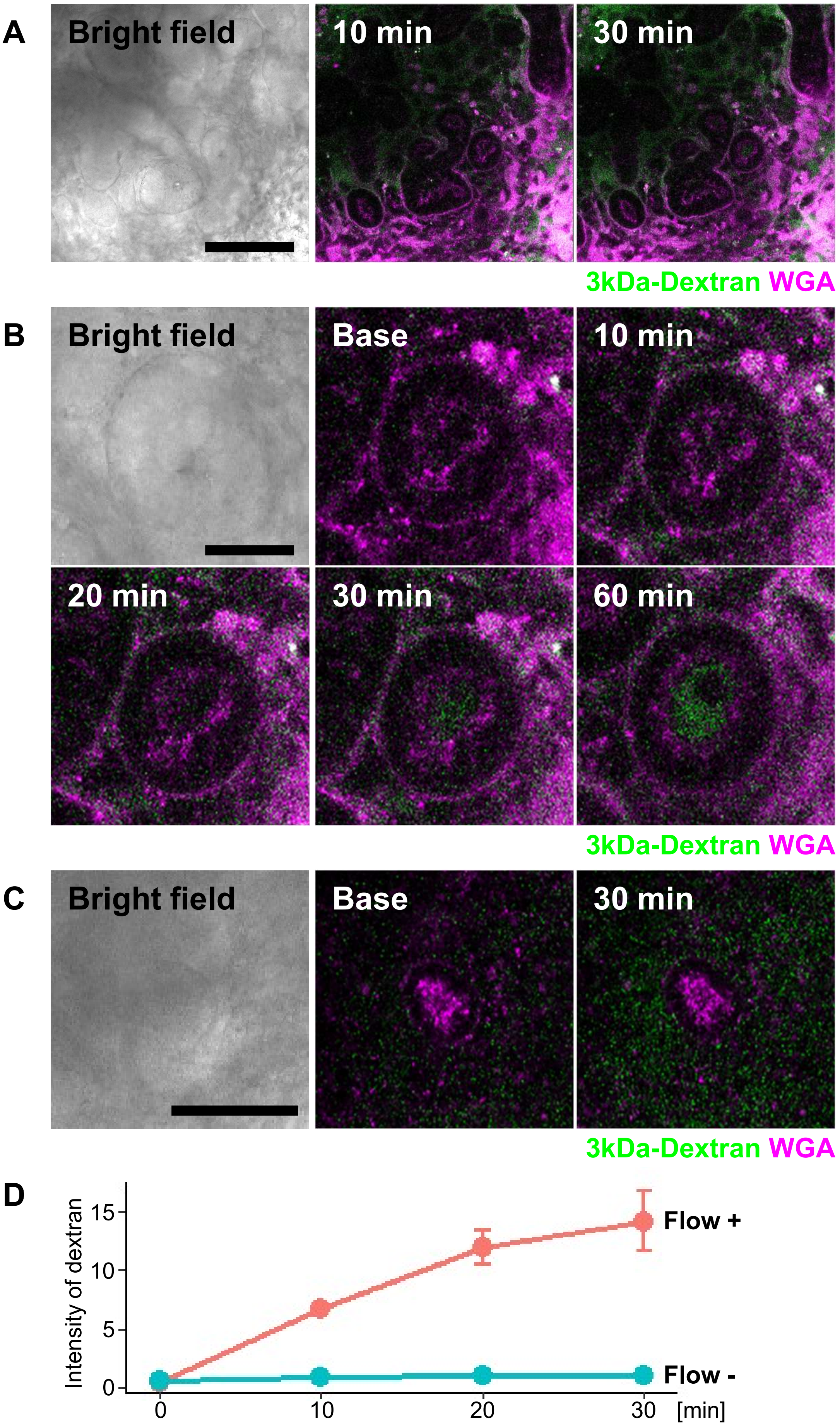

Figure S2: Dextran perfusion imaging under flow conditions and static conditions. The lumens of WGA+ tubules are filled with dextran only in flow condition. A, B) Low and high magnified image in flow condition. Scale bars: 400 µm (A), 100 µm (B). C) High magnified image in static condition. Scale bar: 100 µm. D) Data are shown as mean ± SEM. A linear mixed-effects model with ROI as a random effect demonstrated a significant condition-time interaction.

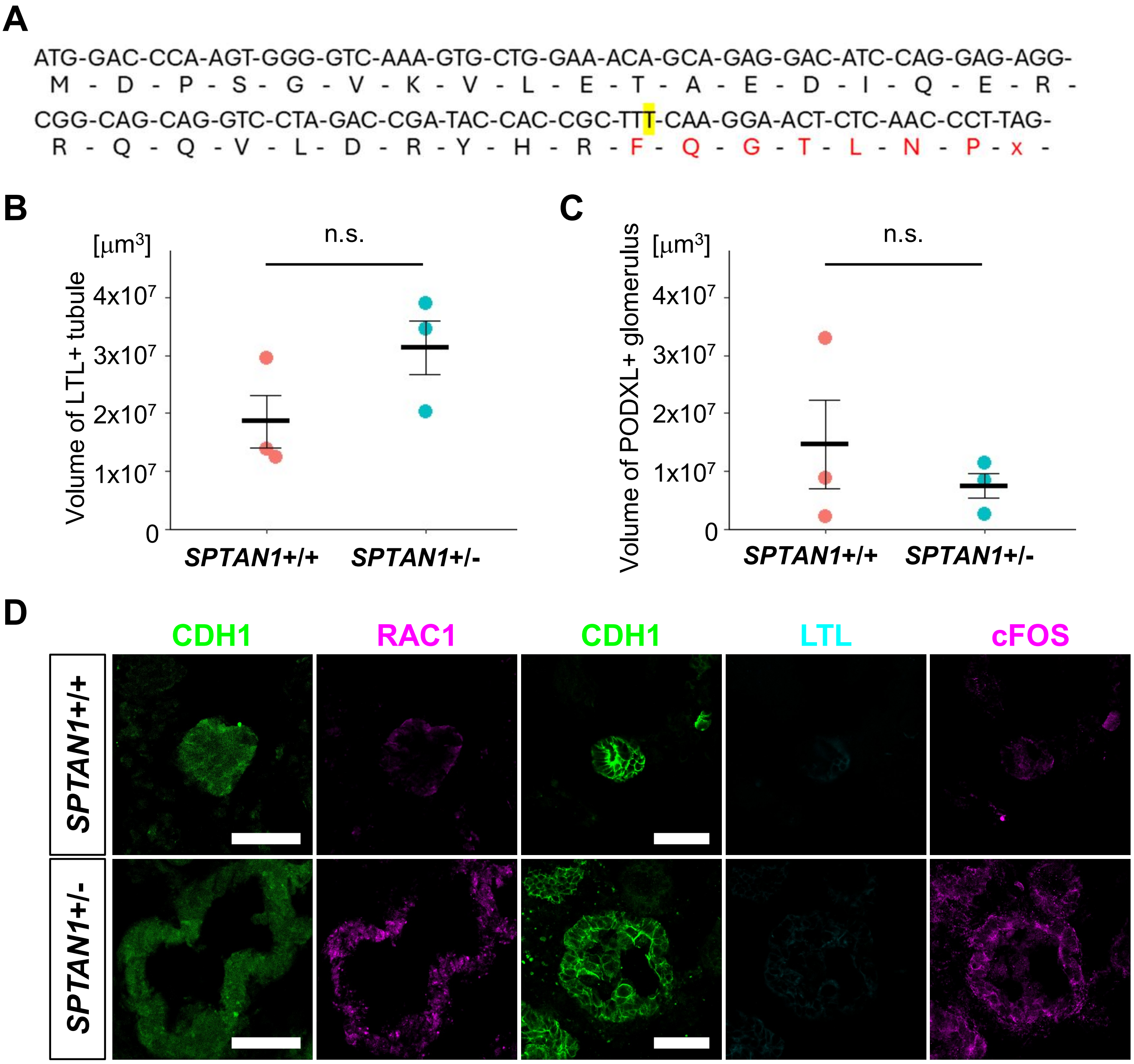

Figure S3: The phenotype caused by SPTAN1 mutations. A) A deep sequencing result of SPTAN1+/- ESCs. Thymine (T) highlighted in yellow has been inserted by CRISPR, causing a frameshift. B,C) Quantification of the total LTL+ tubule volume (B) and PODXL+ glomerular volume (C) in nephron organoids cultured under flow conditions. Each dot represents the average result of a single experiment, which includes 5–6 organoids. Each condition contains 16 organoids from 3 independent experiments. D) Immunostaining for RAC1 and cFOS in SPTAN1+/+ and SPTAN1+/- organoids. Scale bars: 50 μm.

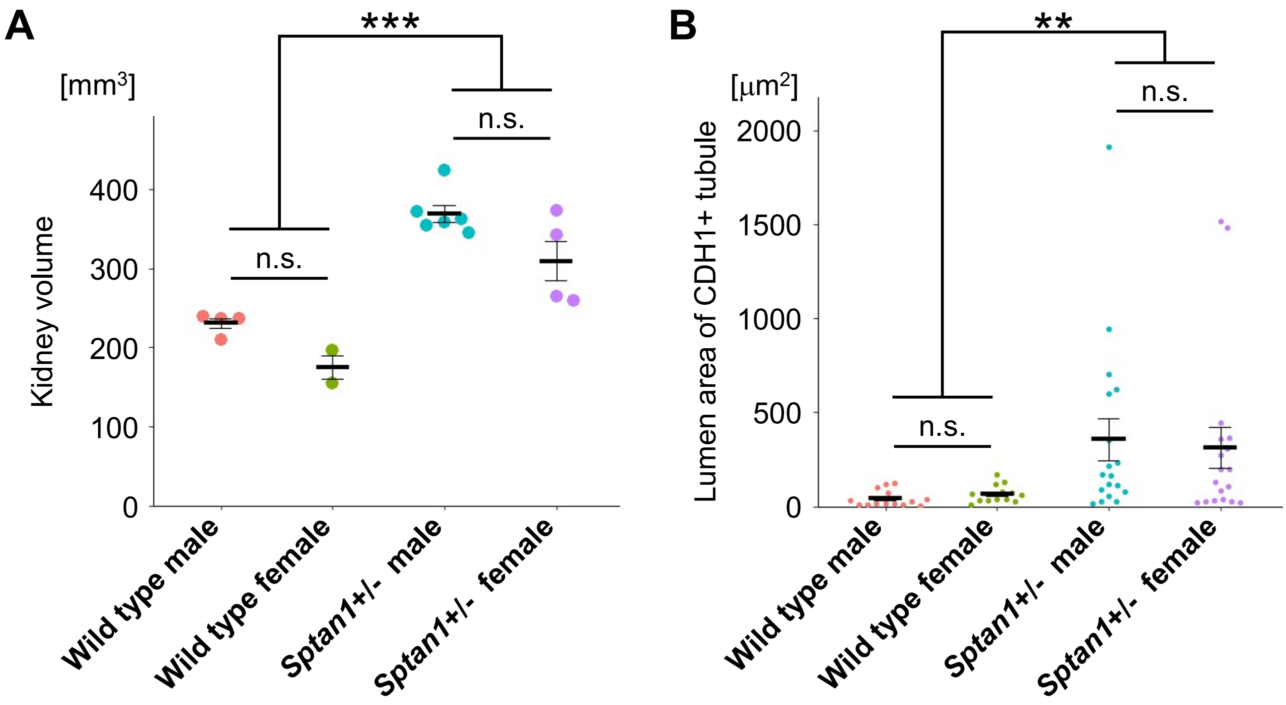

Figure S4: The phenotype caused by SPTAN1 mutations. A) Quantification of the kidney volume in each gender of mice. Each dot represents the volume of a single kidney. ***P < 0.001. B) Quantification of the lumen area in each gender of mice. Each dot represents the measurement of a single tubule. **P < 0.01.

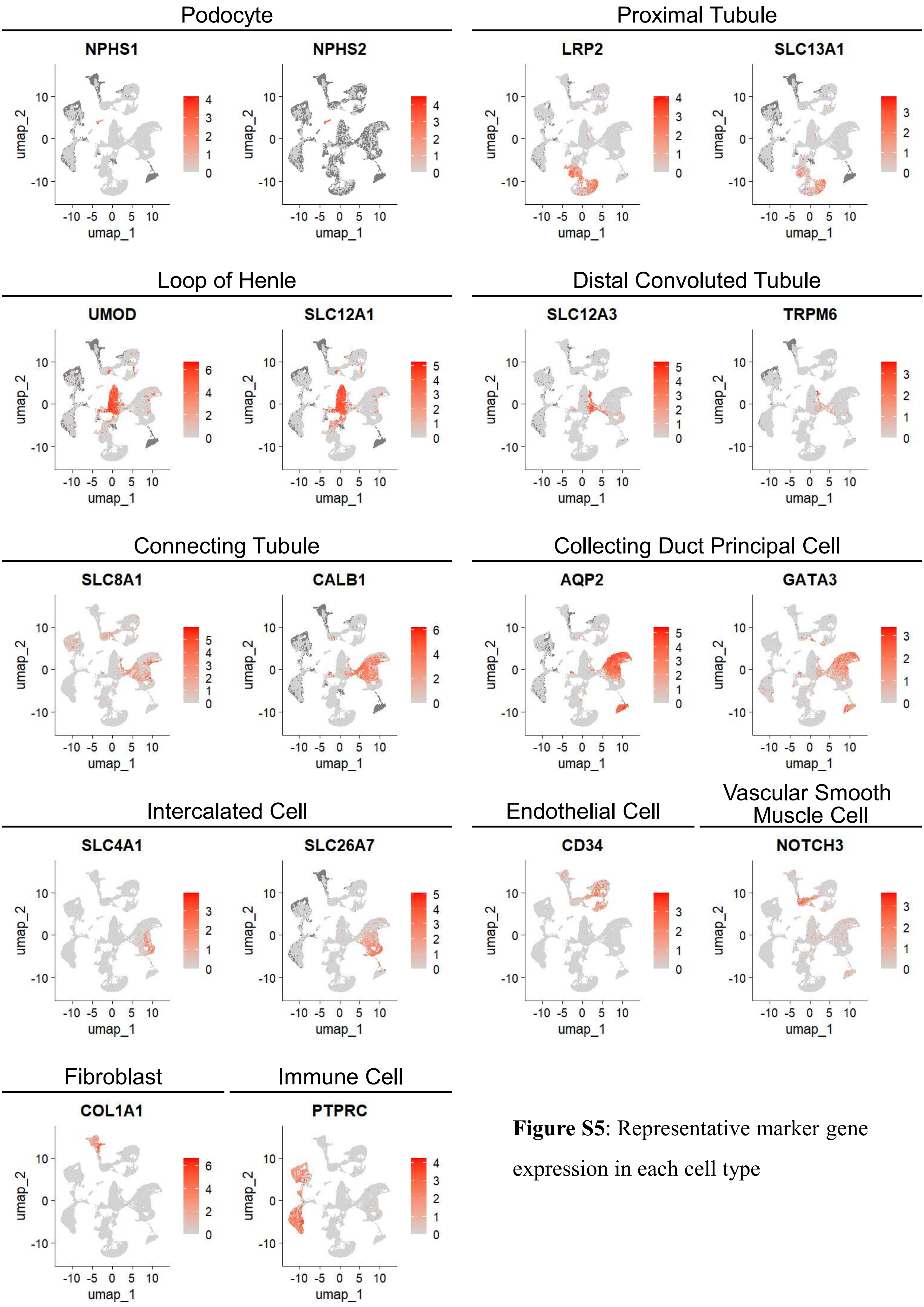

Figure S5: Representative marker gene expression in each cell type

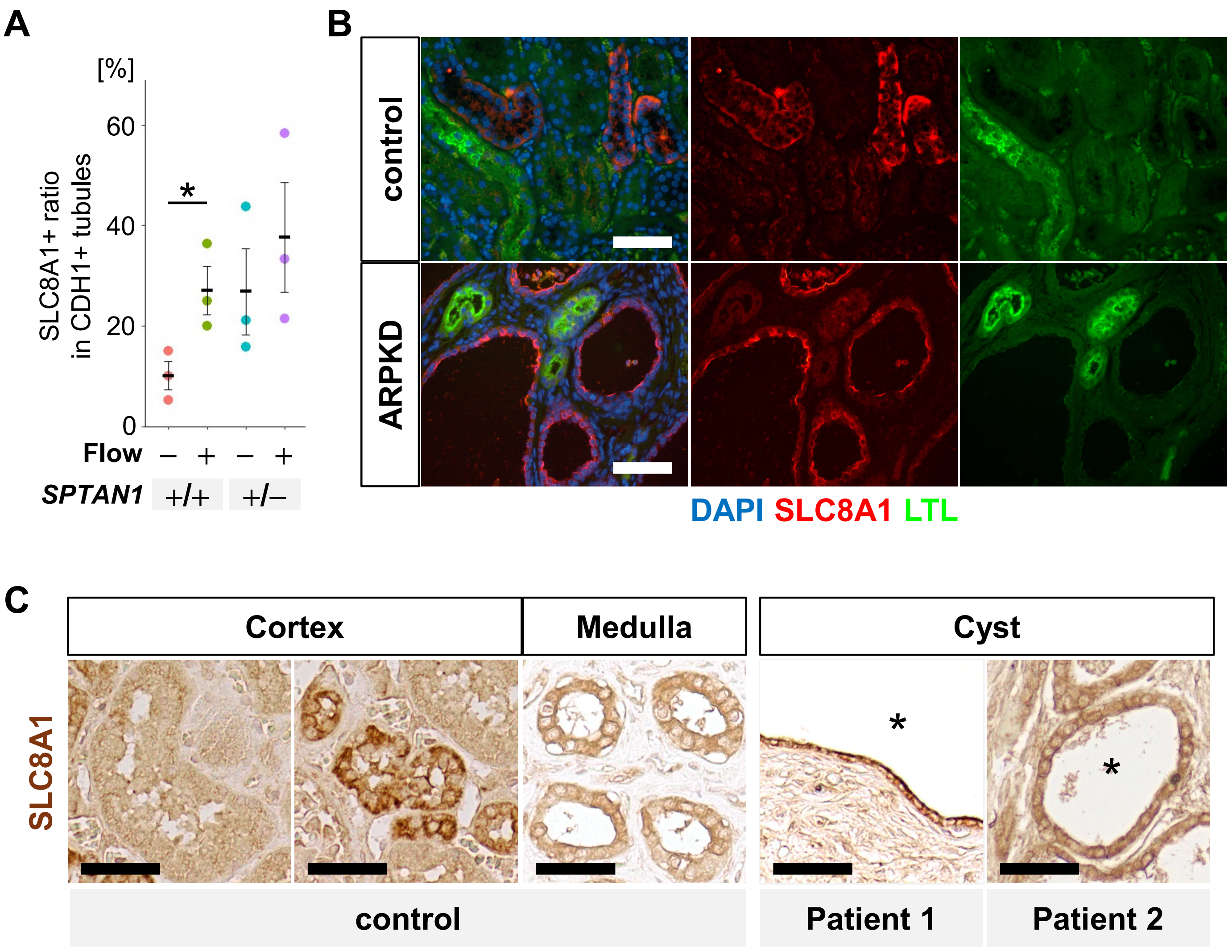

Figure S6: A) Quantification of the SPTAN1 positive percentage in CDH1+ tubules. Each dot represents the mean value of a single organoid. Each condition contains 55, 33, 50 and 43 tubules respectively from 3 organoids. *P < 0.05. B) Immunostaining for SLC8A1 and LTL in human kidneys of healthy control and ARPKD patient. Scale bars: 50 μm. C) Immunohistochemistry for SLC8A1 in human kidneys of healthy control and ARPKD patients. Cortex: SLC8A1 was weakly expressed in the proximal tubules and loop of Henle (left) but strongly expressed in other tubules, including the distal tubules, connecting tubules, and cortical collecting ducts (right). Medulla: The inner medullary collecting ducts exhibited expression levels comparable to those of the proximal tubules. Cyst: In both ARPKD patients, the majority of cysts were positive for SLC8A1, with intensity levels similar to those detected in the cortical distal nephron. Asterisks indicate dilated lumens. Scale bars: 50 μm.

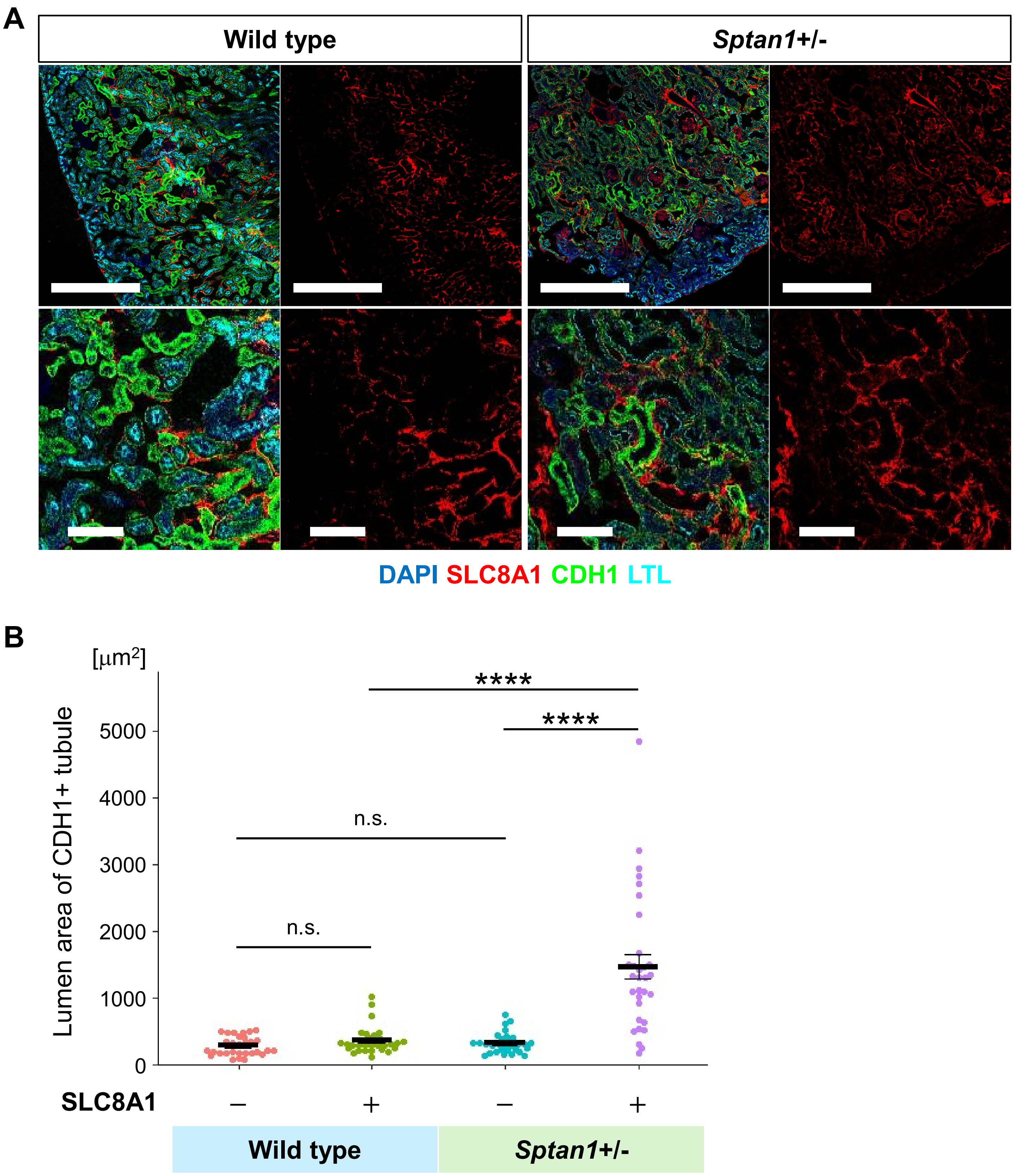

Figure S7: A) Immunostaining for SLC8A1 in mice kidneys of wild type and Sptan1+/-. Scale bars: upper panels, 500 μm; lower panels, 100 μm. B) Quantification of the lumen area in CDH1+ tubules with or without SLC8A1 expression. Each dot represents the measurement of a single tubule. ****$P < 0.0001$

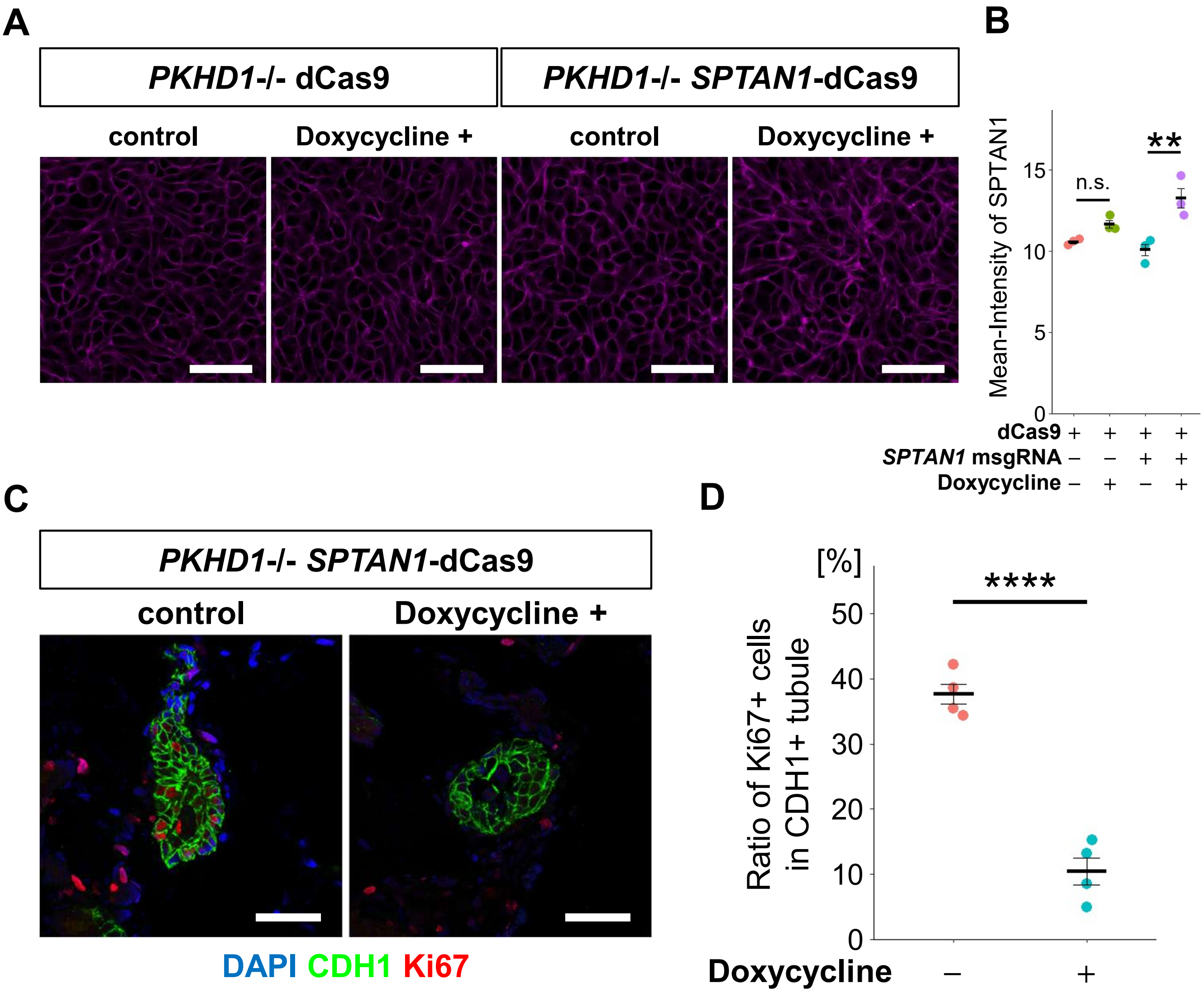

Figure S8: A) Immunostaining for SPTAN1 in PKHD1-/- dCas9 ESCc and PKHD1-/- SPTAN1-dCas9 ESCs. Scale bars: 200 µm. B) Quantification of the SPTAN1 intensity in ESCs. Each dot represents the mean value of a single area. Each condition contains 3 areas selected randomly from plate. **P < 0.01, n.s.: not significant. C) Immunostaining for Ki67 in PKHD1-/- SPTAN1-dCas9 nephron organoids cultured in flow condition on day 35. Scale bars: 50 µm. D) Quantification of the ratio of Ki67 positive cells in CDH1+ tubules. Each dot represents the mean value of a single organoid. ****P < 0.0001.

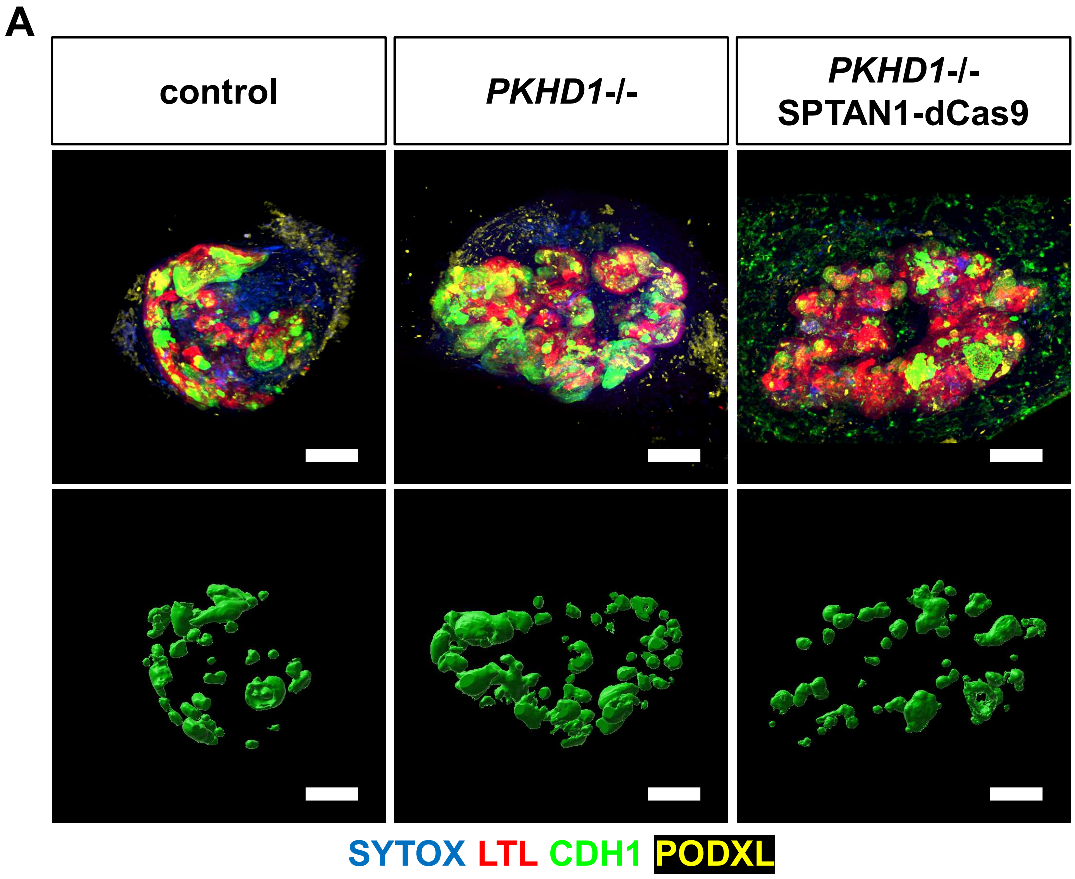

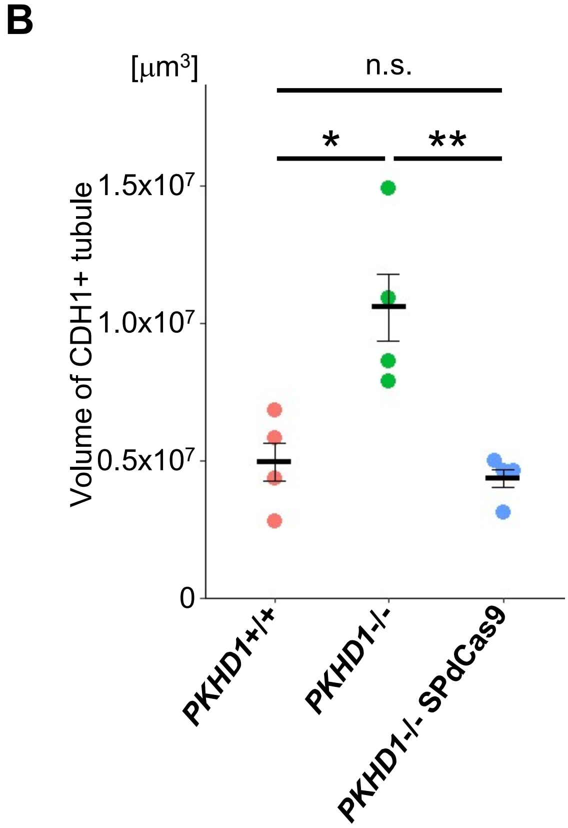

Figure S9: Reproduction of SPTAN1 restoration using iPSC-derived nephron organoids. A) Upper panels: Whole-organoid 3D confocal imaging stacks of PKHD1+/+, PKHD1-/- and PKHD1-/- SPTAN1-dCas9 nephron organoids cultured in flow condition on day 35. In this condition, doxycycline was administered to medium from day 15 to day 35. Lower panels: Imaris surface for CDH1. Scale bars: 200 μm. B) Quantification of the total CDH1+ tubule volume in nephron organoids on day 35. Each dot represents the value of a single organoid. Each condition contains 4 organoids. **P < 0.01, *P < 0.05, n.s.: not significant.
