# Supplementary material for: Deciphering the Impact of RAC1‐SPTAN1 in ARPKD Cystogenesis Using Multifaceted Models
Source: Adv Sci (Weinh). 2026 Feb 26;13(25):e24001. doi: 10.1002/advs.202524001 (PMC13137809; doi:10.1002/advs.202524001)
Supplement: Supplementary file 2 — Supporting File 2: advs74532‐sup‐0002‐DataSet.docx. [file ADVS-13-e24001-s003.docx]

**Supplemental file: The codes for analysis of single cell RNA sequencing**

# Discribe the UMAP from 24 percutaneous renal biopsy samples obtained from healthy humans

library(dplyr)

library(Seurat)

library(patchwork)

# Construct Seurat object from matrix file

sample1.data <- Read10X(data.dir = "C:/R_analysis/scRNAseq/data/file_name")

sample1 <- CreateSeuratObject( counts = sample1.data, project = "sample3k", min.cells = 3, min.features = 200)

sample1[["percent.mt"]] <- PercentageFeatureSet(sample1, pattern = "^MT-")

VlnPlot(sample1, features = c("nFeature_RNA", "nCount_RNA", "percent.mt"), ncol = 3 )

sample1 <- subset(sample1, subset = percent.mt < 30)

sample1 <- NormalizeData(sample1, normalization.method = "LogNormalize", scale.factor = 10000)

# Repeat above code to make 24 Seurat objects (sample1 ~ sample24)

# Describe UMAP after integrating 24 samples (Figure 5A)

sample.normalized <- merge(sample1, y = c(sample2, sample3, sample4, sample5, sample6, sample7, sample8, sample9, sample10, sample11, sample12, sample13, sample14, sample15, sample16, sample17, sample18, sample19, sample20, sample21, sample22, sample23, sample24), add.cell.ids = c("1", "2", "3", "4", "5", "6", "7", "8", "9", "10", "11", "12", "13", "14", "15", "16", "17", "18", "19", "20", "21", "22", "23", "24"), project = "sample1_24", merge.data = TRUE)

sample1_24 <- FindVariableFeatures(sample.normalized, selection.method = "vst", nfeatures = 2000)

all.genes <- rownames(sample1_24)

sample1_24 <- ScaleData(sample1_24, features = all.genes)

sample1_24 <- RunPCA(sample1_24, features = VariableFeatures(object = sample1_24))

sample1_24 <- FindNeighbors(sample1_24, dims = 1:18)

sample1_24 <- FindClusters(sample1_24, resolution = 1.0)

sample1_24 <- RunUMAP(sample1_24, dims = 1:18)

DimPlot(sample1_24, reduction = "umap")

# Verify the expression of any gene on UMAP (Figure S5)

FeaturePlot(sample1_24, features = c("gene_name "), cols = c("lightgrey", "red"))

# Describe the violin plot

library(Seurat)

library(ggplot2)

library(cowplot)

# Extract each nephron segments

cluster_names <- c("2"="LoH", "3"="CD_PC", "5"="PT", "7"="IC","24"="DCT", "26"="CNT", "36"="Po")

subset_seurat$cluster_name <- as.character(subset_seurat$seurat_clusters)

subset_seurat$cluster_name <- factor(subset_seurat$cluster_name, levels = names(cluster_names), labels = cluster_names)

table(subset_seurat$cluster_name)

DimPlot(subset_seurat, reduction = "umap", group.by = "cluster_name")

multiVlnPlot <- function(object, features, pt.size = 0, log = FALSE, title = NULL, out_file = NULL, ...){

scaleFUN <- function(x) sprintf("%.1f", x)

gplot.list <- list()

for(i in 1:length(features)){

vg <- VlnPlot(object = object, features = features[i], pt.size = pt.size, log = log, group.by = "cluster_name", ...)

vg <- vg + scale_y_continuous(labels=scaleFUN)

vg <- vg + scale_fill_discrete(name="Cluster")

vg <- vg + ylab(features[i]) + theme(axis.title.y = element_text(size = 8))

vg <- vg + theme(legend.position = 'none',

axis.title.x = element_blank(),

axis.title.y = element_text(face="bold", vjust = 0.5),

plot.title = element_blank(),

axis.text.x = element_blank(),

axis.text.y = element_text(size = 4),

panel.border = element_rect(colour="gray1", fill=NA) )

gplot.list <- c(gplot.list, list(vg)) }

legend <- get_legend(gplot.list[[1]] +

theme(legend.position = "right") +

guides(color = guide_legend(ncol = 1)) )

if(!is.null(title)){ gtitle <- ggdraw() + draw_label( title, fontface = 'bold', x = 0.5, hjust = 0.5 )

}else gtitle <- NULL

gplot_all <- plot_grid(plotlist = gplot.list, ncol=1)

gplot_all_title <- plot_grid(gtitle, gplot_all, nrow = 2, rel_heights=c(0.5/(length(features)+0.5), length(features)/(length(features)+0.5)) )

gplot_all_legend <- plot_grid(gplot_all_title, legend, ncol=2, rel_widths = c(8, .6))

plot(gplot_all_legend)

if (!is.null(out_file)){ggsave(file=out_file, plot=gplot_all_legend, width=14, height=length(features)+0.5) }

return (gplot_all_legend)}

# Output the figure with marker genes (Figure 5B)

subset_seurat$cluster_name <- factor(subset_seurat$cluster_name, levels = c("Po", "PT", "LoH", "DCT", "CNT", "CD PC","IC"))

segement_markers <- c("PKHD1", "NPHS1", "LRP2", "UMOD", "SLC12A3", "SLC8A1","AQP2", "SLC4A1")

multiVlnPlot(subset_seurat, features = segement_markers, title="markers", out_file = " subset_seurat_ Segment_multiVlnPlot.pdf" )
